# Linkage-specific ubiquitin binding interfaces modulate the activity of the chlamydial deubiquitinase Cdu1 towards poly-ubiquitin substrates

**DOI:** 10.1371/journal.ppat.1012630

**Published:** 2024-10-21

**Authors:** Jan Schlötzer, Alexander Schmalix, Sophie Hügelschäffer, Dominic Rieger, Florian Sauer, Mark D. Tully, Thomas Rudel, Silke Wiesner, Caroline Kisker

**Affiliations:** 1 Institute for Structural Biology, Rudolf-Virchow-Zentrum—Center for Integrative and Translational Bioimaging, University of Würzburg, Würzburg, Germany; 2 Institute of Biophysics and Physical Biochemistry, University of Regensburg, Regensburg, Germany; 3 Department of Microbiology, Biocenter, University of Würzburg, Würzburg, Germany; 4 Structural Biology Group, European Synchrotron Radiation Facility, Grenoble, France; Duke University School of Medicine, UNITED STATES OF AMERICA

## Abstract

The chlamydial deubiquitinase Cdu1 of the obligate intracellular human pathogenic bacterium *Chlamydia trachomatis* plays important roles in the maintenance of chlamydial infection. Despite the structural similarities shared with its homologue Cdu2, both DUBs display remarkable differences in their enzymatic activity towards poly-UB chain substrates. Whereas Cdu1 is highly active towards K48- and K63- poly-UB chains, Cdu2 activity is restricted mostly to mono-UB substrates. Here, we shed light on the molecular mechanisms of the differential activity and the substrate specificity of Cdu1 to better understand the cellular processes it is involved in, including infection-related events. We found that the strikingly elevated activity of Cdu1 relative to its paralogue Cdu2 can be attributed to an N-terminally extended α-helix, which has not been observed in Cdu2. Moreover, by employing isothermal titration calorimetry and nuclear magnetic resonance spectroscopy, we demonstrate the differential recognition of K48- and K63-linked poly-UB substrates by Cdu1. Whereas K63-linked poly-UB substrates appear to be recognized by Cdu1 with only two independent ubiquitin interaction sites, up to four different binding interfaces are present for K48-linked ubiquitin chains. Combined, our data suggest that Cdu1 possesses a poly-UB chain directed activity that may enable its function as a multipurpose DUB with a broad substrate specificity.

## Author summary

As a post-translational modification ubiquitination plays key roles in virtually all cellular processes and is also involved in the host immune response upon infection with intracellular pathogens. *Chlamydia trachomatis* secretes the two deubiquitinase effector proteins Cdu1 and Cdu2, which can subvert the host cell ubiquitin signaling and play crucial roles during infection. Both deubiquitinases have been shown to display drastic differences in their activity towards poly-ubiquitin chains. We here identified the molecular mechanisms by which the enhanced activity of Cdu1 towards these substrates is mediated. Moreover, we discovered marked differences in the recognition of differently linked poly-ubiquitin chains by Cdu1 and mapped binding sites for the individual ubiquitin moieties. Our study thus provides new insights into the enzymatic mechanisms of chlamydial deubiquitinases, which can be crucial to understand their distinct roles during infection.

## Introduction

Ubiquitination is a fundamental post-translational modification in eukaryotic cells and regulates diverse cellular processes including protein degradation, autophagy, cell cycle regulation and DNA damage responses [1–3]. Innate immune responses are also dependent on ubiquitination, as many intracellular pathogen-containing vacuoles or bacterial effector proteins are marked with ubiquitin (UB), targeting them for proteasomal degradation or autophagy [4]. The remarkable diversity and specificity of ubiquitin signaling is achieved by the formation of complex poly-UB structures of different linkages. Ubiquitin can be attached with its carboxy-terminus to lysine residues of target proteins via a cascade of enzymatic reactions that are catalyzed by the concerted activities of the ubiquitin-activating (E1), the ubiquitin-conjugating (E2) and ubiquitin-ligating (E3) enzymes. In addition, ubiquitin itself can be modified at any of its seven lysine residues or at its N-terminal M1 residue to form homotypic, heterotypic, or even branched ubiquitin chains, providing unmatched signaling diversity [5].

Ubiquitin signaling can be reversed by deubiquitinases (DUBs), specialized proteases that hydrolyze isopeptide or M1-linked peptide bonds, thereby removing ubiquitin from the substrate or shortening the ubiquitin chain. DUBs are known to employ a wide range of mechanisms to control substrate specificity, including ubiquitin specific interaction sites. Interfaces that interact with UB/ubiquitin-like (UBl) moieties distal to the cleaved isopeptide position within poly-UB chains are referred to as S-sites, while those that interact with monomers proximal to the cleavage site are termed S’-sites. These sites are then labeled in ascending order according to their position relative to the catalytic center. While UB/UBl selectivity is generally mediated through interactions at the S1-site, substrate or linkage specificity is often achieved via direct interactions with the substrate at the S1’-site [6]. Additional ubiquitin-binding domains in the catalytic core [7,8] or on auxiliary domains [9] further direct the activity of DUBs towards longer, heterotypic, or even branched ubiquitin polymers [10]. The combination of these mechanisms permits a broad spectrum of enzymatic activities, ranging from highly promiscuous DUBs [11] to those with strict substrate selectivity [12,13], with the search for novel ubiquitin binding motifs being an ongoing research topic.

Intracellular bacteria, like *Chlamydia trachomatis* (*C*. *trachomatis*), employ a variety of effector proteins to establish and maintain their replication niche and to evade host cell immune responses. Among the most targeted host signaling pathways are kinase cascades and the ubiquitin-proteasome system [14,15]. Indeed, despite the absence of ubiquitin in bacteria, several intracellular pathogens secrete enzymes that hijack the host ubiquitin system. The obligate intracellular bacterium *C*. *trachomatis*, which is the most common cause of sexually transmitted diseases worldwide and has been classified by the WHO as a human neglected disease pathogen [16], possesses two annotated DUBs, Cdu1 and Cdu2. Infections with *C*. *trachomatis* commonly manifest in the form of conjunctivitis, genitourinary tract infections, and pelvic inflammatory disease as well as trachoma and represent a major burden on health care systems, particularly in the epidemic regions of Southeast Asia and Africa [17,18].

Cdu1 is secreted to the cytoplasmic interface of the chlamydia containing vacuole, which is called the inclusion [19]. Here, its DUB activity has been previously described in the context of various infection-related events. Inclusions of *C*. *trachomatis* strains lacking Cdu1 are highly decorated with K63-linked ubiquitin chains, leading to the recruitment of various autophagy receptors to the inclusion membrane [20]. The anti-apoptotic human protein MCL-1, which is involved in the regulation of apoptosis in Chlamydia-infected cells, is deubiquitinated and stabilized by Cdu1 near the inclusion [19]. Similarly, it was shown that deubiquitination of the inhibitory NF-κB subunit IκBα is promoted in uninfected cells that were transfected with Cdu1, which impairs its degradation and thereby blocks NF-κB activation [21]. Together with Cdu2, Cdu1 has further been linked to the fragmentation of the Golgi apparatus during infection [19,22,23]. In addition to the effects related to its DUB activity, acetylation of several chlamydial effector proteins by Cdu1 has recently been shown to prevent their degradation [23].

**Fig 1:**
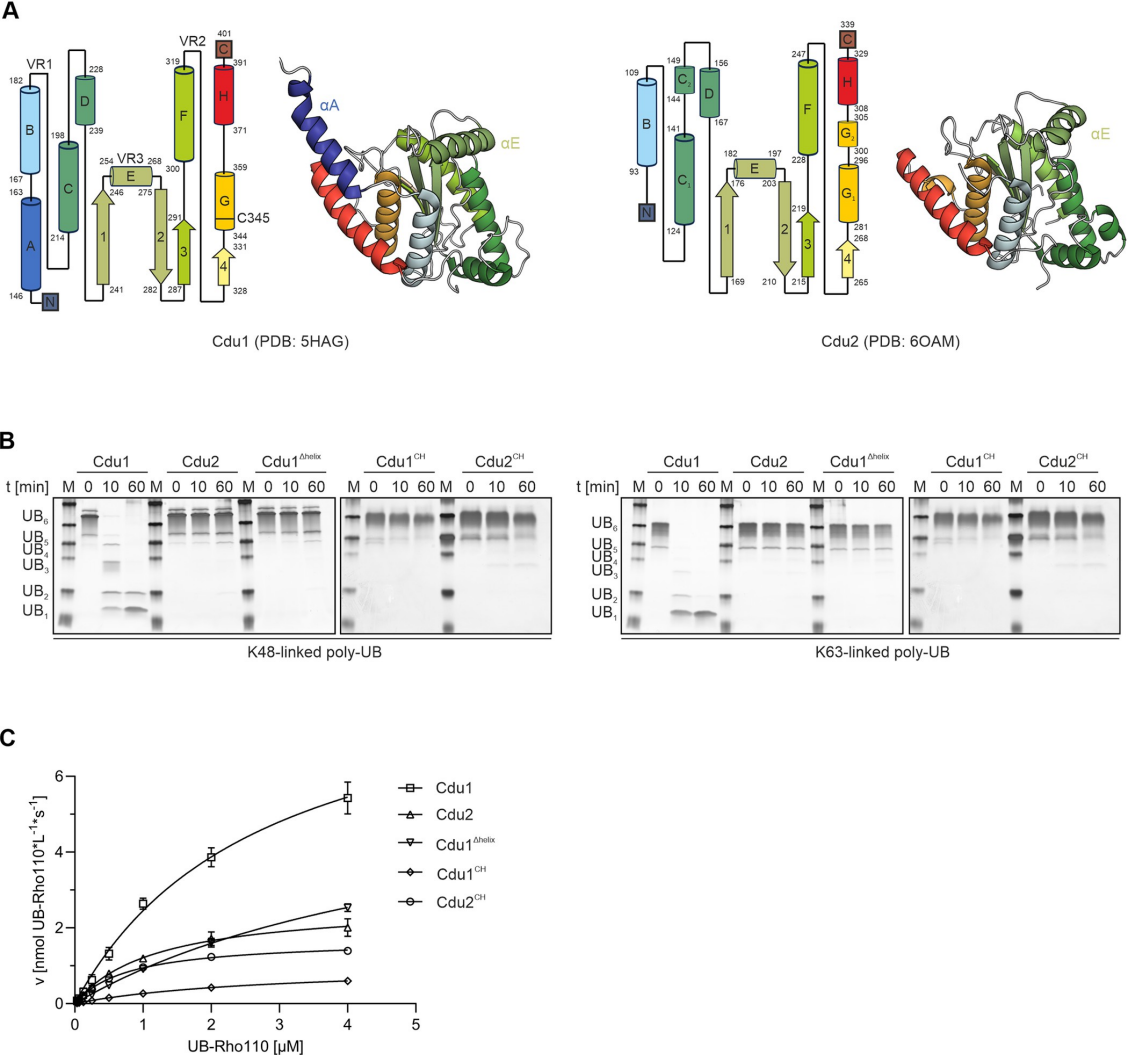
DUB activities and crystal structures of the chlamydial DUBs Cdu1 and Cdu2. **A:** Topology diagrams and crystal structures of Cdu1 (PDB: 5HAG) and Cdu2 (PDB: 6OAM) **B:** Hexa-UB cleavage assay with Cdu1 and Cdu2 variants. 25 nM of the respective DUB variants were incubated at 28°C with 2.5 μM pre-purified hexa-UB substrates. The enzymatic reactions were quenched by the addition of SDS loading buffer at the indicated time points and samples were separated by SDS-PAGE and subsequently silver stained (n = 3). **C:** Michaelis-Menten kinetics of the UB-Rho110Gly cleavage for selected Cdu1 and Cdu2 variants (n = 3). 0.3 nM of the respective Cdu variants were incubated at 28°C with varying concentrations of UB-Rho110Gly and the time dependent hydrolysis was monitored by the increase in fluorescence signal (Extinction: 487 nm; Emission: 535 nm). Error bars smaller than the symbol size are not shown.

Cdu1 belongs to the CE clan of proteases [24], whose members exhibit a variety of substrate specificities, with enzymatic activities ranging from dedicated deubiquitinases and ubiquitin-like proteases to acetyltransferases. Differences in UB/UBl selectivity of these proteases have previously been attributed to three variable regions (VRs) within the otherwise conserved fold of the catalytic domain (Fig 1A). The VRs are involved in the formation of the S1-binding site and mediate extensive interactions with the distal ubiquitin moiety [25]. Cdu1 is the only known member of the CE protease clade that displays dual activity as a DUB with a strong preference towards K48- and K63-linked ubiquitin chains and as an acetyltransferase. Remarkably, the evolutionary closely related Cdu2, which shares its active site architecture with Cdu1 (Fig 1A), does not exhibit acetyl transferase activity and has been reported to be substantially less active towards (poly-)UB substrates in vitro compared to Cdu1 [22,26].

To provide insights into the molecular mechanisms by which Cdu1 facilitates its enhanced activity towards poly-UB substrates, we performed an exhaustive biophysical analysis of Cdu1. Its mode of action and specificity towards different substrates may provide insights into the differential roles of the two DUBs during the infection process. We established the importance of an N-terminal α-helix in Cdu1 for its elevated activity towards ubiquitin chains and further demonstrated its differential recognition of K48- and K63-linked poly-UB substrates. While Cdu1 can interact with K48-linked poly-UB chains with up to four distinct ubiquitin binding interfaces, only two of these interfaces are available for K63-linked substrates. These additional ubiquitin binding motifs steer Cdu1’s activity towards specific isopeptide bond positions within K48-linked poly-UB chains, whereas K63-linked substrates are hydrolyzed without a clear preference for selected isopeptide bond positions.

## Results

### The DUB activity of Cdu1 towards poly-UB chain substrates is modulated by its N-terminal alpha helix

In 2016, Pruneda et al. characterized three variable regions within the conserved fold of CE clan proteases that facilitate substrate recognition and specificity by shaping the S1-binding site. Crystal structures of Cdu1 demonstrated that VR3 in Cdu1 is formed by an inserted α-helix (helix αE in Fig 1A) that is unique to chlamydial DUBs among the characterized CE clan proteases and functions as an important interaction interface with the distal ubiquitin [25]. Cdu2 had previously been shown to be significantly less active in cleaving poly-UB chains than Cdu1 but retained comparable activity towards the mono-UB mimetic DUB substrate UB-Rho110Gly [26].To elucidate the molecular mechanisms by which the drastic increase in activity of Cdu1 towards poly-UB substrates is facilitated, we compared the existing crystal structure of the Cdu1 catalytic domain (PDB: 6GZT) to the one of the closely related Cdu2 (PDB: 6MRN). Both enzymes are structurally highly conserved, which is reflected by an rmsd value of 0.88 Å when superimposed. Analyses of important residues of their S1-site architectures have previously been performed [22,26] but a conclusive explanation for the enhanced activity of Cdu1 towards poly-UB chains could not be provided. One striking difference in the available crystal structures of Cdu1 and Cdu2 is the presence of an extended N-terminal alpha helix (helix αA) in Cdu1 (Fig 1A). As the corresponding residues had been omitted in the construct of Cdu2 that was used for crystallization, we consulted secondary structure prediction by JPred [27] and models generated by Alphafold [28] of Cdu2 to establish the presence of this α-helix in Cdu2 (S1A and S1B Fig). Both methods indicated that this feature was either absent or predicted with low confidence and distorted in Cdu2.

To assess the importance of helix αA on the activity of Cdu1, we performed cleavage assays with K48- and K63-linked poly-UB chains and conducted Michaelis-Menten kinetics with the mono-UB mimetic substrate UB-Rho110Gly in the presence and absence of helix αA. Cdu1 was highly efficient in hydrolyzing both K48- and K63-linked hexa-UB substrates, albeit with a preference for K63-linked over K48-linked poly-UB chains (Fig 1B) as has been previously reported [29]. In contrast, Cdu2 as well as an N-terminally truncated variant of Cdu1 (Cdu1^Δhelix^), which lacks helix αA, were virtually inactive in vitro. Interestingly, this dramatic reduction in activity was not reflected in Michaelis-Menten kinetics performed with the mono-UB mimetic substrate UB-Rho110Gly (Fig 1C). While deletion of helix αA resulted in a moderately reduced activity of Cdu1 (v_max_ of 6.3 nmol*L^-1^*s^-1^ for Cdu1^Δhelix^ vs. 9.2 nmol*L^-1^*s^-1^ for Cdu1; Table 1), the differences were minor compared to the drastic reduction in activity observed with poly-UB substrates. Concurrently, replacement of the N-terminal helix αA of Cdu1 by the corresponding amino acids of Cdu2 (Cdu1^CH^) was not sufficient to restore the activity towards hexa-UB or UB-Rho110Gly (Fig 1B and 1C and Table 1). Furthermore, a chimeric variant of Cdu2 harboring the N-terminal α-helix of Cdu1 (Cdu2^CH^) did not exhibit increased activity compared to wildtype Cdu2. Analysis of protein integrity by differential scanning fluorimetry (DSF) confirmed that the overall stability of the Cdu variants was not affected by the variations at the N-terminus (S2A Fig), further supporting the important but not exclusive role of helix αA for the cleavage of poly-UB chains.

**Table 1:** Kinetic parameters of UB-Rho110Gly cleavage by selected Cdu variants.

	Cdu1	Cdu2	Cdu1^Δhelix^	Cdu1^CH^	Cdu2^CH^
v_max_ [nmol UB-Rho110*L^-1^*s^-1^]	9.2 (± 0.6)	2.6 (± 0.3)	6.3 (± 0.1)	1.0 (± 0.1)	1.7 (± 0.001)
K_M_ [μmol*L^-1^]	2.8 (± 0.08)	1.2 (± 0.1)	5.9 (± 0.3)	2.9 (± 0.2)	0.8 (± 0.1)
k_cat_/K_M_ [μmol^-1^*L*s^-1^]	11.1 (± 1.0)	7.4 (± 1.2)	3.6 (± 0.2)	1.2 (± 0.2)	7.1 (± 0.7)
R^2^	0.99	0.99	0.99	0.99	0.99

### The interaction of Cdu1 with poly-ubiquitin substrates is linkage specific and dependent on helix αA

**Fig 2:**
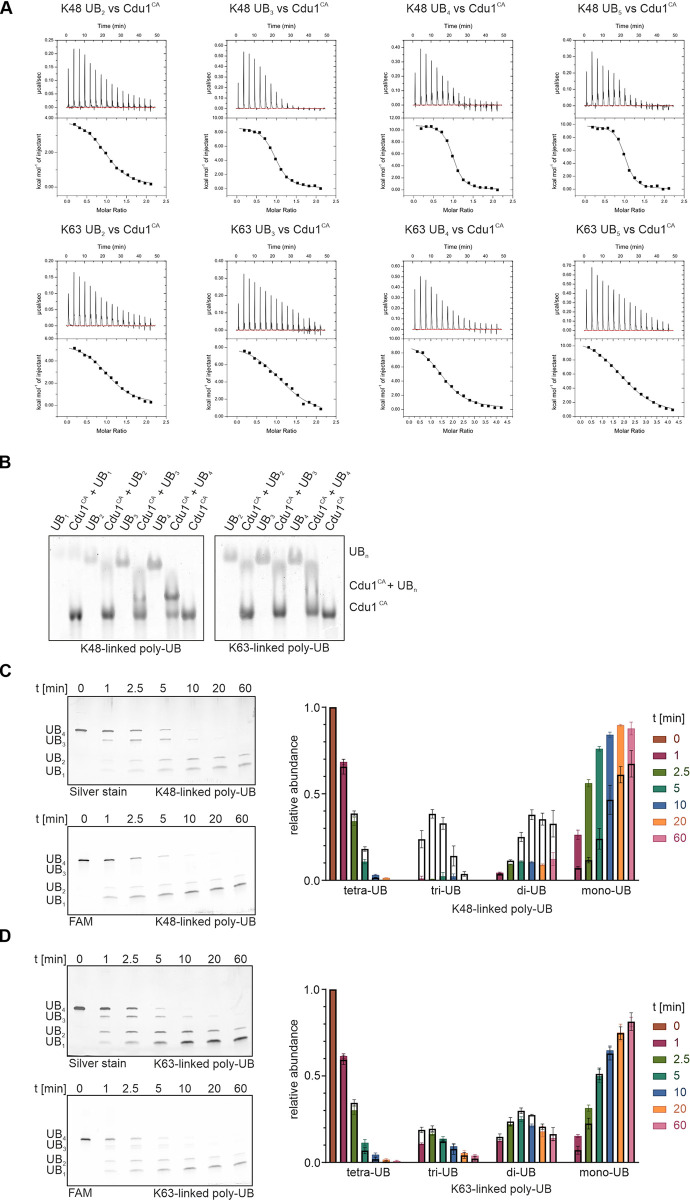
Differential recognition of poly-UB substrates modulates DUB activity in Cdu1. **A:** Representative thermograms and isotherms for ITC measurements of Cdu1^CA^ with K48- or K63-linked poly-UBs. 200 μM Cdu1^CA^ was titrated to 20 μM of either K48-linked (*top*) and K63-linked (*bottom*) poly-UB chains ranging from di-UB to penta-UB (n = 3). **B:** Native PAGE based band shift experiments with pre-formed complexes of 100 μM Cdu1^CA^ and 50 μM K48-linked poly-UBs (*left*) from mono-UB to tetra-UB and 50 μM K63-linked (*right*) di-UB to tetra-UB (n = 2). **C & D:** FAM-labeled poly-UB chain cleavage assay with 5 μM K48-linked (**C**) and K63-linked (**D**) tetra-UB substrates that were distally labeled with fluorescein. *Upper panel*: Silver staining. *Lower panel*: Fluorescence signal. For the quantification (*right diagram*), colored bars indicate the fraction of fluorescently labeled product and the black bordered bars indicate the fraction of total ubiquitin at each individual time point (n = 3).

To understand the activity modulating role of helix αA, we performed isothermal titration calorimetry (ITC) experiments in which isolated K48- and K63-linked poly-UB substrates of varying chain length were titrated with catalytically inactive Cdu1^CA^. While we could not detect any interaction between Cdu1^CA^ and mono-UB at the initially employed concentrations, interactions with longer poly-UB substrates of either linkage were readily observed by ITC (Fig 2A and Table 2). In contrast, Cdu1^CA Δhelix^ was restricted from binding to poly-UB substrates similar to a variant of Cdu1 that included the two mutations I225A and I267R, which have been previously described to abrogate DUB activity by blocking the S1 binding site [22] (S3A and S3B Fig). While we did detect some residual binding enthalpies for longer substrates with Cdu1^CA Δhelix^ and minor exothermic interactions for K48-linked but not K63-linked chains with Cdu1 I225A/I267R, the affinities of both variants to the various poly-UB substrates were too low to unambiguously fit accurate binding parameters. Consequently, helix αA is not only fundamental for the activity of Cdu1 towards poly-UB substrates but also participates in the interaction with poly-UB chains.

**Table 2:** Binding parameters for the interactions of Cdu1^CA^ with poly-UB substrates.

			K_D_ [μmol*L^-1^]	N	ΔH [cal*mol^-1^]	ΔS [cal*mol^-1^*deg^-1^]
WT		mono-UB	6.91 (± 1.46)	0.81 (± 0.02)	1663 (± 116)	29.2 (± 0.7)
	K48	di-UB	2.58 (± 0.75)	1.02 (± 0.12)	3674 (± 813)	37.8 (± 2.2)
		tri-UB	0.80 (± 0.09)	1.01 (± 0.05)	7052 (± 1457)	51.3 (± 4.6)
		tetra-UB	0.31 (± 0.04)	0.99 (± 0.05)	9627 (± 1129)	61.8 (± 3.6)
		penta-UB	0.28 (± 0.09)	0.99 (± 0.14)	10013 (± 756)	63.4 (± 1.9)
	K63	di-UB	2.52 (±0.08)	0.92 (±0.15)	5989 (± 796)	45.6 (± 2.0)
		tri-UB	3.01 (±0.31)	1.24 (±0.21)	7345 (± 1200)	49.6 (± 4.0)
		tetra-UB	4.47 (±0.62)	1.60 (±0.14)	9933 (± 535)	57.4 (± 2.0)
		penta-UB	6.56 (± 0.03)	2.30 (± 0.22)	10895 (± 982)	60.0 (± 3.1)
M149A/M153A	K48	di-UB	2.91 (±0.33)	0.98 (±0.03)	1890 (±101)	31.6 (±0.6)
		tri-UB	1.78 (±0.48)	1.01 (±0.08)	6399 (±805)	48.0 (±0.5)
		tetra-UB	1.24 (±0.62)	0.95 (±0.03)	10910 (±70)	63.0 (±0.8)
L294R/N296G	K48	di-UB	n.d.	n.d.	n.d.	n.d.
		tri-UB	n.d.	n.d.	n.d.	n.d.
		tetra-UB	n.d.	n.d.	n.d.	n.d.
Q384A/A385R	K48	di-UB	3.44 (±0.06)	0.96 (±0.05)	3886 (±694)	37.9 (±2.3)
		tri-UB	1.65 (±0.33)	1.04 (±0.02)	7698 (±1245)	52.1 (±3.8)
		tetra-UB	1.42 (±0.15)	1.14 (±0.06)	7860 (±1073)	52.9 (±3.8)

For Cdu1^CA^, all interactions with poly-UB chains were endothermic and driven by a strong entropic component (Table 2). Interestingly, we further observed a differential recognition of K48- and K63-linked poly-UB substrates. For K48-linked ubiquitin substrates a progressively stronger affinity of Cdu1^CA^ in a chain length-dependent manner was obtained, with calculated dissociation constants ranging from 2.58 μM for di-UB, 800 nM for tri-UB and up to 310 nM for tetra-UB. Titrations with even longer K48-linked poly-UBs exceeding tetra-UB, however, did not result in tighter associations (here shown for penta-UB). The interactions with all K48-linked ubiquitin substrates could be consistently fitted with monovalent binding stoichiometries, which indicated the simultaneous interaction of Cdu1 with up to four individual ubiquitin moieties of a single poly-UB chain. The concerted interactions of these distinct ubiquitin binding interfaces with individual ubiquitin molecules of longer chain substrates thus likely facilitate the increasingly stronger interaction towards poly-UB substrates in an avidity-like manner.

In contrast, a similar effect could not be shown with K63-linked poly-UB substrates (Table 2). While we observed an increased affinity of K63-linked di-UB compared to mono-UB, lengthening the chain of K63-linked poly-UB substrates beyond di-UB did not enhance the interaction with Cdu1^CA^. Instead, the determined dissociation constants stagnated in the low micromolar range and higher order binding stoichiometries were observed with longer substrates (Fig 2A and Table 2). We therefore likely observed the simultaneous saturation of elongated K63-linked poly-UB chains with multiple Cdu1 molecules.

Poly-UB chains of different linkage are thought to adopt distinct conformations in solution. K63-linked chains are rather elongated, while K48-linked chains form extensive intra-chain interactions resulting in a compact ubiquitin arrangement. To exclude that the observed differences in the interactions are solely mediated by the alternative conformations of the two chain types, we further analyzed the activity and binding affinities of Cdu1 towards M1-linked (extended conformation) and K11-linked (compact conformation) poly-UB substrates (S4A and S4B Fig and S1 Table). Neither linear M1-linked nor K11-linked tetra-UB chains were hydrolyzed efficiently by Cdu1 under our assay conditions (S4B Fig). Concurrently, we observed no interaction of Cdu1 with M1-linked tetra-UB by ITC (S4A Fig; top left panel). K11-linked poly-UB chains, in contrast, were recognized by Cdu1 with similar affinities to those determined for K48- and K63-linked di-UB substrates but importantly, compared to K48-linked chains, the affinity of the interaction with K11-linked poly-UBs did not improve in a chain length dependent manner (S4A Fig and S1 Table vs. Table 2). Combined, the strict substrate selectivity and the distinct substrate recognition pattern of Cdu1, strongly support the presence of defined and linkage specific ubiquitin interaction interfaces.

To obtain binding parameters with mono-UB, we repeated the ITC experiments with substantially increased concentrations. In this case, 200 μM Cdu1^CA^ were titrated with 2 mM mono-UB. The resolved interaction was endothermic and resulted in a 1:1 stoichiometry with a calculated K_D_ of ~7 μM (S3C Fig and Table 2), which was above that measured for all di-UB substrates.

The chain length dependent formation of Cdu1^CA^ complexes with poly-UB substrates was further assessed by gel shift assays using native PAGE. Whereas pre-formed complexes of Cdu1^CA^ with mono-UB and K48-linked di- and tri-UB were visibly disrupted during gel electrophoresis, complexes of K48-linked tetra-UB with Cdu1^CA^ remained stable (Fig 2B), supporting the chain length dependent effect observed in ITC. In contrast, no Cdu1^CA^ complexes could be resolved with K63-linked poly-UB substrates, independent of the chain length used for assembly. The lack of additional substrate binding motifs for K63-linked chains, as anticipated from our ITC experiments, may not have permitted stable complex association within the gel. Importantly, this effect was independent of the apparent increase in the concentration of available ubiquitin monomers in equimolar samples of longer poly-UB chain substrates. This was evident as complexes with different poly-UB chains which were formed at substrate stoichiometries mimicking the apparent mono-UB concentration of the tetra-UB samples were still separated to the same extent during gel electrophoresis (S4C Fig). Taken together, our ITC and gel shift assays established the differential recognition of K48- and K63-linked poly-UB substrates by Cdu1. While K48-linked chains are recognized by up to four individual ubiquitin binding sites, for K63-linked chains only two such interaction sites are available.

### The differential binding sites of Cdu1 modulate its activity towards K48- and K63-linked ubiquitin chains

To test whether the distinct binding modes for K48- and K63-linked ubiquitin chains steer the hydrolase activity of Cdu1 towards specific isopeptide linkage positions, we prepared a set of custom poly-UB substrates, which were exclusively and site-specifically labeled with fluorescein (FAM) at the most distal ubiquitin moiety of the substrate chain. When we performed the poly-UB chain cleavage assay with these fluorescently labeled substrates and quantified the relative abundance of fluorescent and native poly-UB product species, we observed marked differences in the hydrolysis of K48- and K63-linked poly-UB substrates (Fig 2C and 2D). Incubation of K63-linked FAM-labeled poly-UB substrates with Cdu1 resulted in a uniform distribution of fluorescently labeled product chains (Fig 2D). This indicates a site independent, stochastic hydrolysis of isopeptide bonds within K63-linked poly-UB chains by Cdu1 and was consistent for labeled tetra- and penta-UBs (S5B Fig). In contrast, a distinct cleavage pattern was observed with K48-linked poly-UB substrates (Figs 2C and S5A) of all chain lengths, for which we observed a significant overrepresentation of fluorescent mono- and to a lesser extend di-UB compared to longer poly-UB chain products. Importantly, modification with the FAM-label did not impact the interaction of the substrate with Cdu1, as confirmed by ITC (S5C Fig and S1 Table). This result indicates that the additional binding sites of Cdu1 for K48-linked poly-UB chains likely steer its activity towards distal isopeptide bond positions with K48-linked but not with K63-linked poly-UB chains.

### Low resolution reconstruction of the Cdu1 complexes with poly-UB substrates by SEC-SAXS

**Fig 3:**
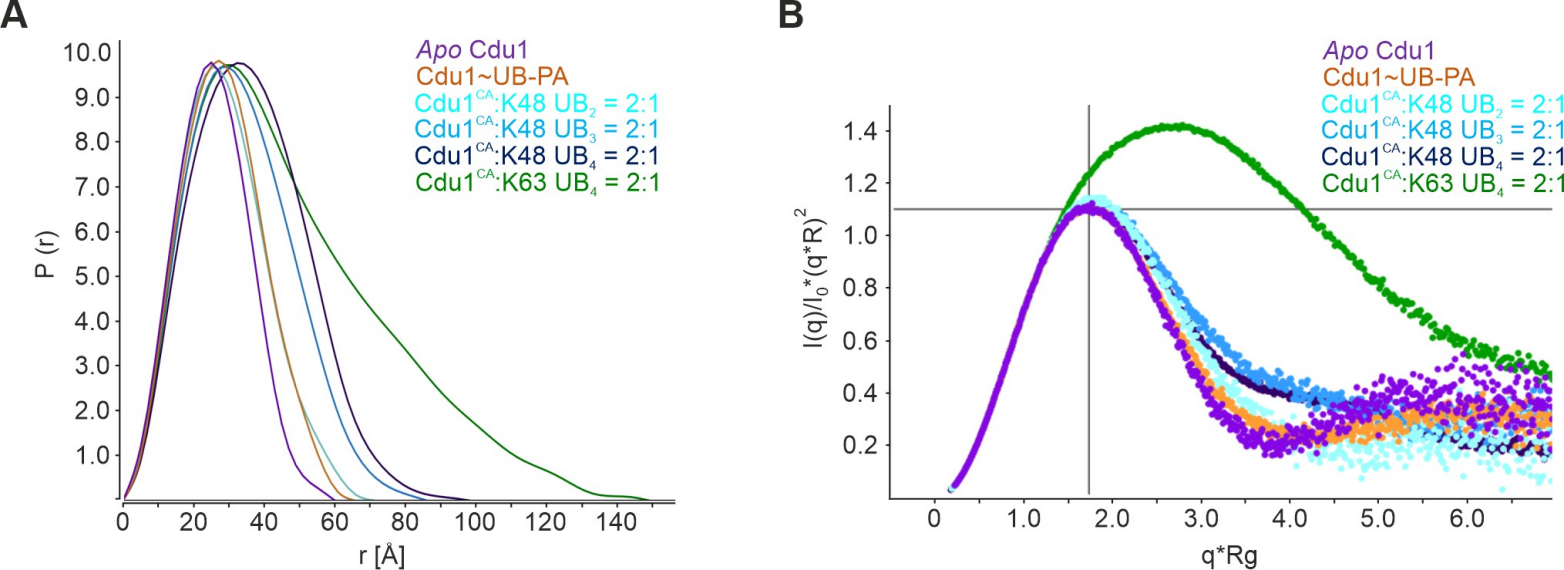
SEC-SAXS experiments of Cdu1 complexes with K48- and K63-linked poly-UB chains. **A:** Calculated P(r) distributions of SEC-SAXS data generated with apo Cdu1 (magenta), Cdu1 covalently modified with UB-PA (orange), and Cdu1^CA^ complexes pre-formed with either K48-linked di- (cyan), tri- (blue) and tetra-UB (dark blue) or K63-linked tetra-UB (green), respectively. **B:** Kratky-Plot for the SEC-SAXS samples defined in (**A**).

To further define the stoichiometry of the interaction between differentially linked poly-UB substrates and Cdu1, we performed size-exclusion chromatography coupled to small angle X-ray scattering (SEC-SAXS) experiments (Fig 3 and S2 Table) with Cdu1^CA^ and ubiquitin chains of varying length. Apo Cdu1^CA^ adopted a spherical conformation in solution with a radius of gyration (R_g_) of 20.09 Å and a maximum diameter (D_max_) of 60 Å, which correlated well (χ^2^ of 1.28) with simulated data generated from the published crystal structures (Fig 3A).

Cdu1 covalently modified at the S1-site with ubiquitin-propargylamide (UB-PA) or complexes of Cdu1^CA^ formed with K48-linked poly-UB substrates displayed a steady chain length dependent increase in the R_g_ and D_max_ values while maintaining their spherical shape (Table 3), consistent with a tighter association of the progressively longer ubiquitin chains with an increasingly extended interaction surface of Cdu1. In contrast, complexes formed with K63-linked tetra-UB were much larger in size and adopted an elongated shape (Fig 3A and Table 3). This was particularly evident by the shifted maximum in the Kratky-plot and suggested that substantial regions of the K63-linked tetra-UB were not involved in the interaction with Cdu1^CA^ (Fig 3B). Importantly, SAXS data obtained for the isolated poly-UB chains, i.e. in the absence of Cdu1^CA^ differed significantly from those obtained for the various Cdu1 complexes (S6A and S6B Fig).

**Table 3:** Fitted SAXS parameters.

	Cdu1	Cdu1 + UB-PA	Cdu1^CA^ + K48 di-UB	Cdu1^CA^ + K48 tri-UB	Cdu1^CA^ + K48 tetra-UB	Cdu1^CA^ + K63 tetra-UB
R_g_ [Å]	20.09 (+/- 0.08)	22.17 (+/- 0.07)	22.5 (+/- 0.13)	25.98 (+/- 0.13)	27.95 (+/- 0.10)	37.84 (+/- 0.36)
Volume [Å^3^]	44*10^3^	58*10^3^	56*10^3^	83*10^3^	109*10^3^	117*10^3^
D_max_ [Å]	60.0	65.5	71.0	86.0	96.0	149.0
MW [kDa]	29	39	38	55	72	81
theoretical MW [kDa]	31	40	49	57	66	66

For Cdu1^CA^ complexes with K48-linked poly-UBs, molecular weights calculated using the Scatter software [30], coincided with the theoretical values of a constant 1:1 binding stoichiometry (Table 3). However, the calculated molecular weight of the complex formed with the K63-linked tetra-UB indicated partial higher order complex assemblies. Similarly, low resolution *ab initio* models of all data sets confirmed the globular nature of Cdu1^CA^ complexes with K48-linked ubiquitin chains in contrast to the elongated shape predicted from data obtained with the K63-linked tetra-UB complex (S6C Fig).

### Monomeric ubiquitin interacts with the S1-site of Cdu1

**Fig 4:**
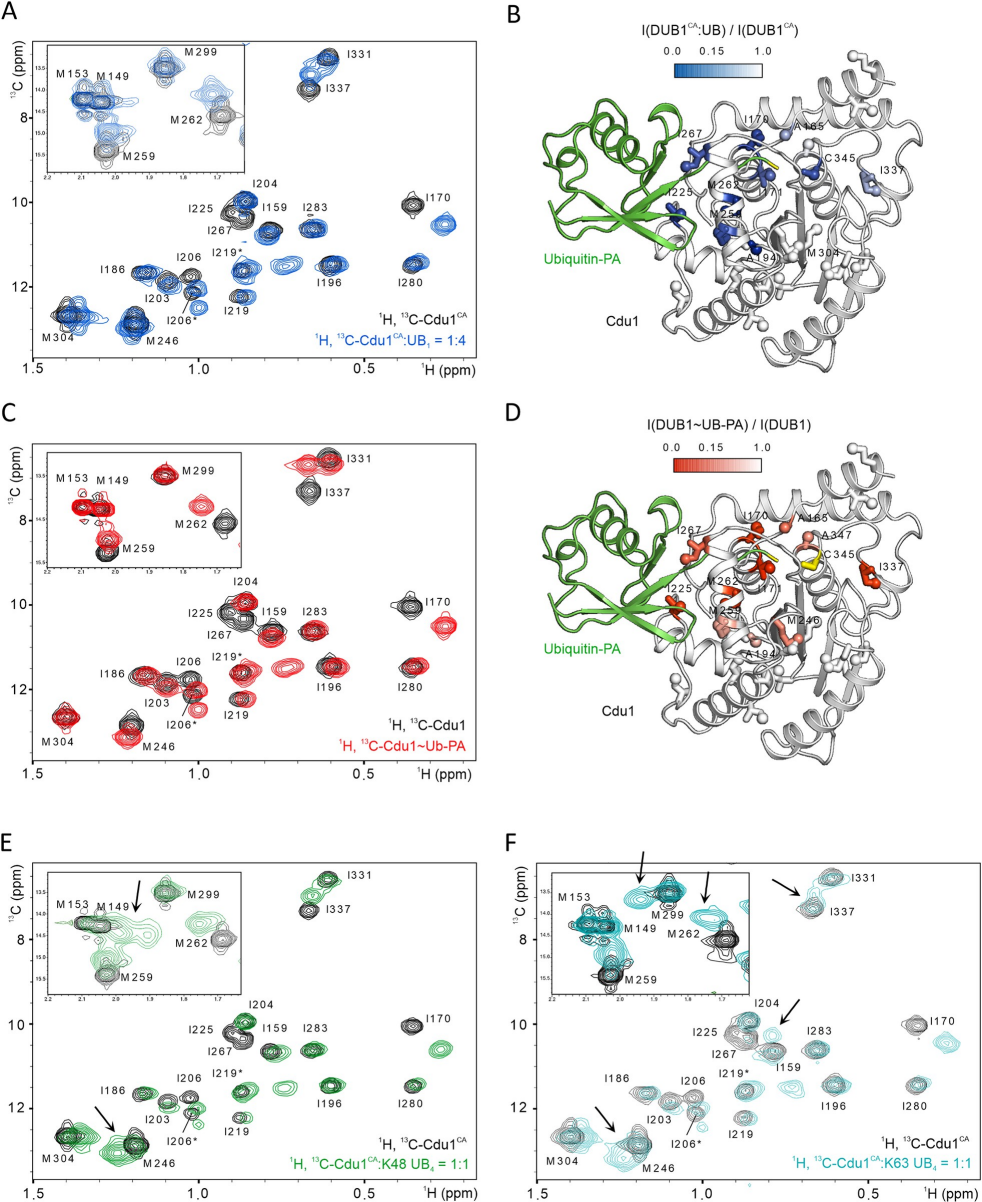
NMR titration of Cdu1 with K48- and K63-linked poly-UB chains. **A:** Overlay of selected regions of the 2D ^1^H,^13^C-correlation spectra of AILMV-labeled Cdu1^CA^ in the absence (black) and in the presence of a 4-fold excess of monomeric ubiquitin (blue). **B:** Mapping of the CSPs on the structure of Cdu1 modified with UB-PA (PDB-ID: 6GZS). Cdu1 residues for which an intensity loss of more than 85% was observed in **(A)** are highlighted with different shades of blue depending on their relative change in intensity as indicated, while the conjugated ubiquitin is depicted in green. Ala, Ile, and Met side chains are shown as sticks, while the isotope labeled carbon groups are shown as spheres. **C:** As in **(A)**, but for apo Cdu1 (black) and Cdu1~UB-PA (red). **D:** As in **(B)**, but with Cdu1 residues color coded by the intensity loss observed in **(C)** with different shades of red as indicated. **E & F**: As **(A)**, but for apo Cdu1^CA^ (black) and equimolar concentrations of K48-linked (green) **(E)** or K63-linked (cyan) **(F)** tetra-UB chains. Peak splitting upon poly-UB binding is highlighted with black arrows, while K48- and K63 specific CSPs are indicated with green and cyan arrows, respectively.

To gain more insights into the differential interaction of Cdu1 with K48- and K63-linked poly-UB chains, we conducted solution state nuclear magnetic resonance (NMR) experiments with ^1^H,^13^C-methyl labeled Cdu1 (Fig 4). We titrated per-deuterated Cdu1^CA^ specifically ^1^H,^13^C-labeled at the Ala β-, Ile δ_1_-, Met ε-, Leu δ- and Val γ-methyl positions (AILMV) with unlabeled monomeric ubiquitin or K48- and K63-linked tetra-UB. To map the chemical shift perturbations (CSPs) caused by ligand binding to the structure of Cdu1 we assigned all Ile and Met and some of the Ala methyl resonances by mutagenesis and 3D NOE spectroscopy. Interestingly, two spatially close Ile residues, I206 and I219, possess two δ_1_-methyl resonances frequencies (Fig 4A) demonstrating that they each occupy two distinct conformations in solution.

Comparison of the 2D ^1^H,^13^C-methyl-HMQC NMR spectra of apo Cdu1^CA^ and Cdu1^CA^ titrated with mono-UB revealed that numerous resonances (A165, I170, I171, A194, I225, M259, M262, I267, I337, A345, and A347) were exchange broadened upon addition of sub-stoichiometric amounts of monomeric ubiquitin and re-appeared at distinct resonance frequencies at later points of the titration (Fig 4A). This behavior is typical for a chemical exchange process on the slow NMR time scale that is often associated with binding affinities in the nanomolar to low micromolar range.

To map the CSPs observed in the NMR spectra to the 3D structure of Cdu1 we quantified the intensity loss upon ligand binding (I/I_ref_) for all assigned methyl groups. Consistent with the crystal structures of Cdu1 covalently linked to UB-PA [22,31], most of the Cdu1^CA^ peaks that were affected by mono-UB binding belonged to amino acids of the S1-site (Fig 4B). This included A345 that corresponds to the catalytic Cys in the wildtype protein, M259 and M262 within the prominent helix αE, as well as I225 and I267. The mutation of the latter two residues to Gly and Arg, respectively, had previously been shown to interfere with Cdu1 DUB activity [22]. In addition, we observed CSPs for residues neighboring the S1-site or the catalytic Cys (I170, I171, A194 and A347) and for I337 that is located adjacent to the catalytic Cys and within the margins of a putative S1’-ubiquitin binding site.

To untangle allosteric effects induced by binding of ubiquitin to the S1-site from potential additional binding sites, we occupied the S1-site with covalently bound mono-UB (Cdu1^WT^~UB-PA) and repeated the NMR experiments (Fig 4C and 4D). Of note, the spectra of the WT and inactive Cdu1^CA^ were almost identical except for the expected disappearance of the A345 resonance in the WT spectrum and small resonance changes of residues in immediate vicinity of the catalytic C345 (Fig 4C). This demonstrates that the cysteine to alanine mutation that inactivates DUB activity in Cdu1 does not induce significant structural rearrangements in the S1-site. While all CSPs observed for mono-UB can be explained by the interaction of UB-PA with the S1-site, subsequent titration of Cdu1^WT^~UB-PA with mono-UB resulted in no additional changes of the spectrum (S7 Fig). This indicates that additional ubiquitin binding sites on Cdu1 are too weak to be detected by NMR with monomeric ubiquitin and are likely only occupied during the interaction with poly-UB chains.

### Poly-UB binding induces additional chemical shift changes in Cdu1

We next investigated how Cdu1^CA^ may discriminate between K48- and K63-linked ubiquitin chains. For the NMR titration of Cdu1^CA^ with K48- and K63-linked tetra-UB chains small, but significant differences occurred compared to the titration with mono-UB (Fig 4E and 4F). In particular, several resonances including M246, M259 and M262 experienced slight peak splitting upon addition of poly-UB chains indicating mixed occupancies with distinct individual ubiquitin moieties within the chains. Moreover, K48-, but not K63-linked poly-UB, induced gradually increasing chemical shift changes of M149 and M153 in the N-terminally extended helix αA that plays a role in poly-UB hydrolysis (Fig 4E). In contrast, for K63-linked poly-UB we detected a peak splitting for M299 and I337 that was absent for K48-linked poly-UB (Fig 4F). Together these differences in CSPs for the differentially linked poly-UB chains support the distinct binding modes of K48- and K63 chains observed in the experiments described above (Figs 2 and 3 and Tables 2 and 3). Our NMR analyses thus show minor, but linkage-specific changes in the Cdu1^CA^ NMR spectra when comparing the binding of K48- and K63-linked poly-UB chains.

### Mutation of residues specific for the recognition of poly-UB substrates disrupts the progressive increase in affinity upon chain elongation

**Fig 5:**
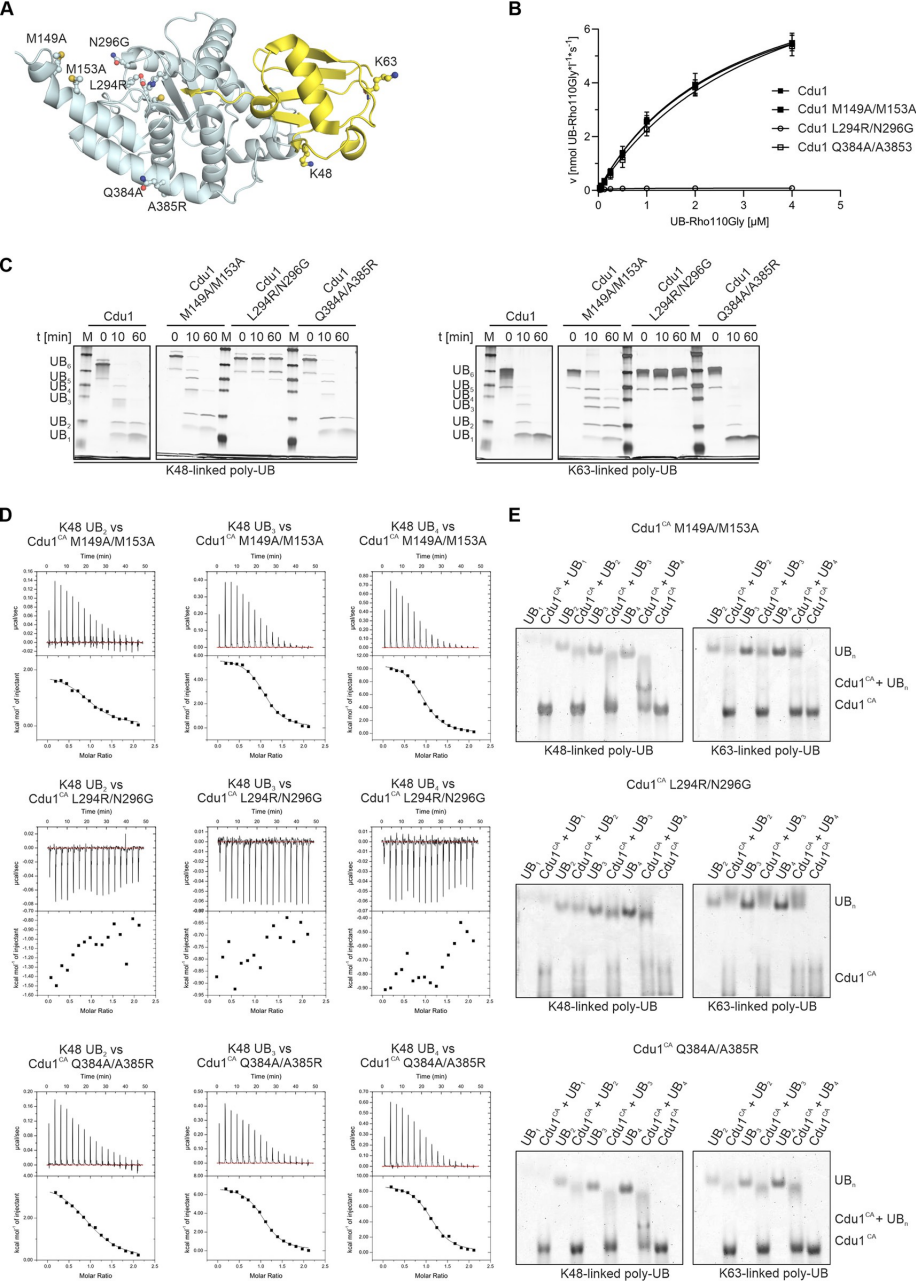
Mutation of residues specific for the recognition of poly-UB substrates disrupt the progressive increase in affinity upon chain elongation. **A:** Location of the mutated residues shown in ball-and-stick representation in the structure of Cdu1 covalently linked to UB-PA (PDB-ID: 6GZS). **B:** Michaelis-Menten kinetics for the UB-Rho110Gly cleavage of selected Cdu1 variants. (n = 3). Error bars smaller than the symbol size are not shown. **C:** Hexa-UB cleavage assay with Cdu1 variants. DUB variants (25 nM) were incubated at 28°C with 2.5 μM pre-purified hexa-UB. The enzymatic reactions were quenched by the addition of SDS loading buffer at the indicated time points and samples were separated by SDS-PAGE and subsequently silver stained (n = 2). **D:** ITC measurements of Cdu1^CA^ variants with K48-linked poly-UB chains. 300 μM Cdu1^CA^ variants were titrated to 30 μM K48-linked poly-UBs ranging from di-UB to tetra-UB (n = 3) **E:** Native PAGE experiments with complexes of the selected Cdu1 variants with mono-UB and K48-linked (*left*) or K63-linked (*right*) poly-UB chains (n = 2).

Having identified regions in Cdu1 that play a role in the linkage-specific recognition of poly-UB chains by NMR spectroscopy, we introduced mutations at positions that may determine selectivity and tested their effects on enzymatic activity and substrate association (Fig 5A). In the L294R/N296G variant two residues in the putative S1’-site were mutated. These residues are located between M299 and I337 that both exhibited chemical shift changes specific for K63-linked poly-UB (Fig 4F). The M149A/M153A variant lacked the two methionine residues that displayed specific CSPs upon addition of K48-linked poly-UB during the NMR titration, while the Q384A/A385R variant targeted helix αH that is situated close to K48 of the distal ubiquitin in the S1-site and adjacent to I170 and A347 that displayed strong CSPs in the NMR titrations (Fig 4). While the M149A/M153A and Q384A/A385R variants displayed wildtype-like activity in the UB-Rho110Gly based assay, the activity of the L294R/N296G variant was drastically reduced (Fig 5B and Table 4). This effect was independent of protein integrity as all variants were stable and properly folded as determined by differential scanning fluorimetry and circular dichroism spectroscopy (S2 Fig). Similarly, the L294R/N296G variant was only marginally active towards hexa-UB substrates, whereas the Q384A/A385R variant exhibited wildtype-like activity. Intriguingly, while the M149A/M153A variant displayed diminished activity towards K48-linked hexa-UB, its ability to cleave K63-linked chains was also impaired (Fig 5C). M149 and / or M153 therefore participate not only in the recognition of K48- but also K63-linked substrates, presumably within the S1’-site, despite no clear evidence for such an interaction in our NMR data.

**Table 4:** Kinetic parameters of UB-Rho110Gly cleavage by Cdu1 variants.

	WT	M149A/M153A	L294R/N296G	Q384A/A385R
v_max_ [nmol UB-Rho110*L^-1^*s^-1^]	9.2 (± 0.6)	9.5 (± 0.9)	0.1 (± 0.005)	10.8 (± 2.2)
K_M_ [μmol*L^-1^]	2.8 (± 0.08)	2.9 (± 1.1)	0.2 (± 0.02)	4.0 (± 1.8)
k_cat_/K_M_ [μmol^-1^*L*s^-1^]	11.1 (± 1.0)	10.9 (± 5.0)	1.7 (± 0.3)	9.1 (± 6.0)
R^2^	0.99	0.99	0.99	0.99

Catalytically inactive versions of these Cdu1 variants were also impaired in their ability to bind K48-linked poly-UB chains. An increase in complex stability of the L294R/N296G variant with longer chained K48-linked substrates comparable to the wildtype enzyme could not be detected by native PAGE and no association to poly-UB chains was observed by ITC (Fig 5D and 5E and Table 2), confirming the drastic effects of these mutations on substrate association and turnover. Both the M149A/M153A and the Q384A/A385R variants formed less stable complexes with K48-linked poly-UB chains, as complex formation could only be observed for tetra-UB and was in general visibly weaker in native PAGE (Fig 5E vs. 2B). In addition, ITC experiments with both constructs revealed a diminished affinity towards K48-linked poly-UB substrates compared to wild-type Cdu1 (Table 2). In particular, the improvement in binding affinity when comparing K48-linked tri- and tetra-UB was significantly reduced for both variants. Interestingly, the increase in binding enthalpy that we measured between complexes formed with K48-linked tri- and tetra-UB and wildtype Cdu1, respectively, could also be observed for Cdu1 M149A/M153A but not with the Cdu1 Q384A/A385R variant (Fig 5D and Table 2).

Finally, we investigated the effects of these mutations on the isopeptide bond selectivity of Cdu1’s activity towards FAM-labeled poly-UB substrates (S8 Fig). Like wildtype Cdu1, all tested variants hydrolyzed K63-linked tetra-UB in a stochastic manner with equally likely cleavage at each isopeptide bond position. Much to our surprise, however, both Cdu1 M149A/M153A and Cdu1 Q384A/A385R still displayed the same cleavage selectivity pattern for K48-linked tetra-UB as observed for the wildtype construct, with fluorescent tri-UB being strongly underrepresented compared to fluorescent di- and mono-UB species. Notably, similar to native poly-UB substrates the activity towards K48- and K63-linked chains was reduced with respect to the M149A/M153A variant and more pronounced for K63-linked substrates, i.e. indicating a small but noticeable effect of helix αA on K63-linked poly-UB cleavage. For the Q384A/A385R variant we observed a drastic reduction in its hydrolysis of K48-linked but not K63-linked FAM-labeled poly-UB substrates. While a minor reduction may also be observed with native chains, the effect was nowhere as apparent as with the FAM-labeled substrates.

### Deletion of helix αA or mutations at the S1’-site lead to impaired IκBα stabilization

**Fig 6:**
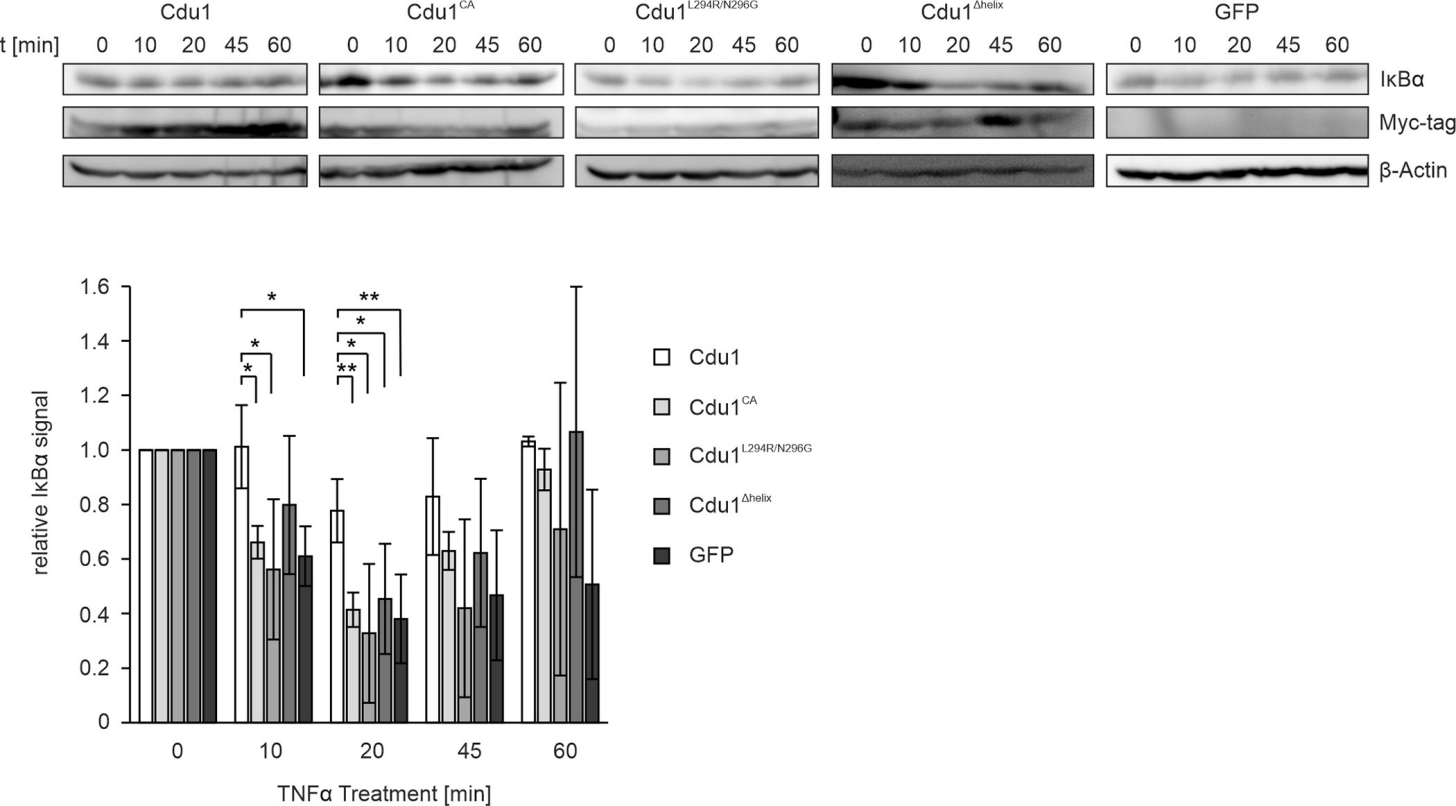
Deletion of helix αA or disruption of the S1’-site in Cdu1 reduce the DUB-dependent IκBα stabilization in TNFα treated HEK293T cells. HEK293T cells were transfected with the respective Myc-tagged Cdu1 variants or GFP controls and treated with 30 ng/ml recombinant human TNF-α for the indicated time frames. The Cdu1 dependent IκBα stabilization was analyzed in the cell lysates by western blot analysis (n = 3). The signal intensities were quantified with ImageJ and the statistical significance was calculated using a one-sided t-test (*: p < 0.05, **: p < 0.01).

To determine if our in vitro observations have an impact in the cellular context, we adopted the protocol established by Le Negrate et al. [21] and analyzed the effect of Cdu1 and its variants towards IκBα degradation in the presence of TNFα (Fig 6). HEK293T cells were co-transfected with selected Cdu1 variants, and IκBα degradation was induced by the addition of TNFα. As previously reported, IκBα levels in control cells, here transfected with GFP, decreased significantly within 20 minutes post-stimulation and only began to recover after 60 minutes. However, the effect of TNFα on IκBα abundance was less pronounced in our experiments compared to previous studies [21]. Nevertheless, transfection with WT Cdu1 resulted in a clear stabilization of IκBα following TNFα treatment, compared to the controls. In contrast, cells transfected with the Cdu1^Δhelix^ or the Cdu1 L294R/N296G variants displayed a generally reduced stabilization of IκBα, similar to the results observed with the catalytically inactive Cdu1^CA^ variant. The deletion of helix αA or the disruption of the S1’-site via the L294R and N296G mutations in Cdu1 thus not only interfered with the interaction and activity towards poly-UB substrates in vitro but also resulted in the reduced stabilization of IκBα, one of the key cellular targets of Cdu1.

## Discussion

**Fig 7:**
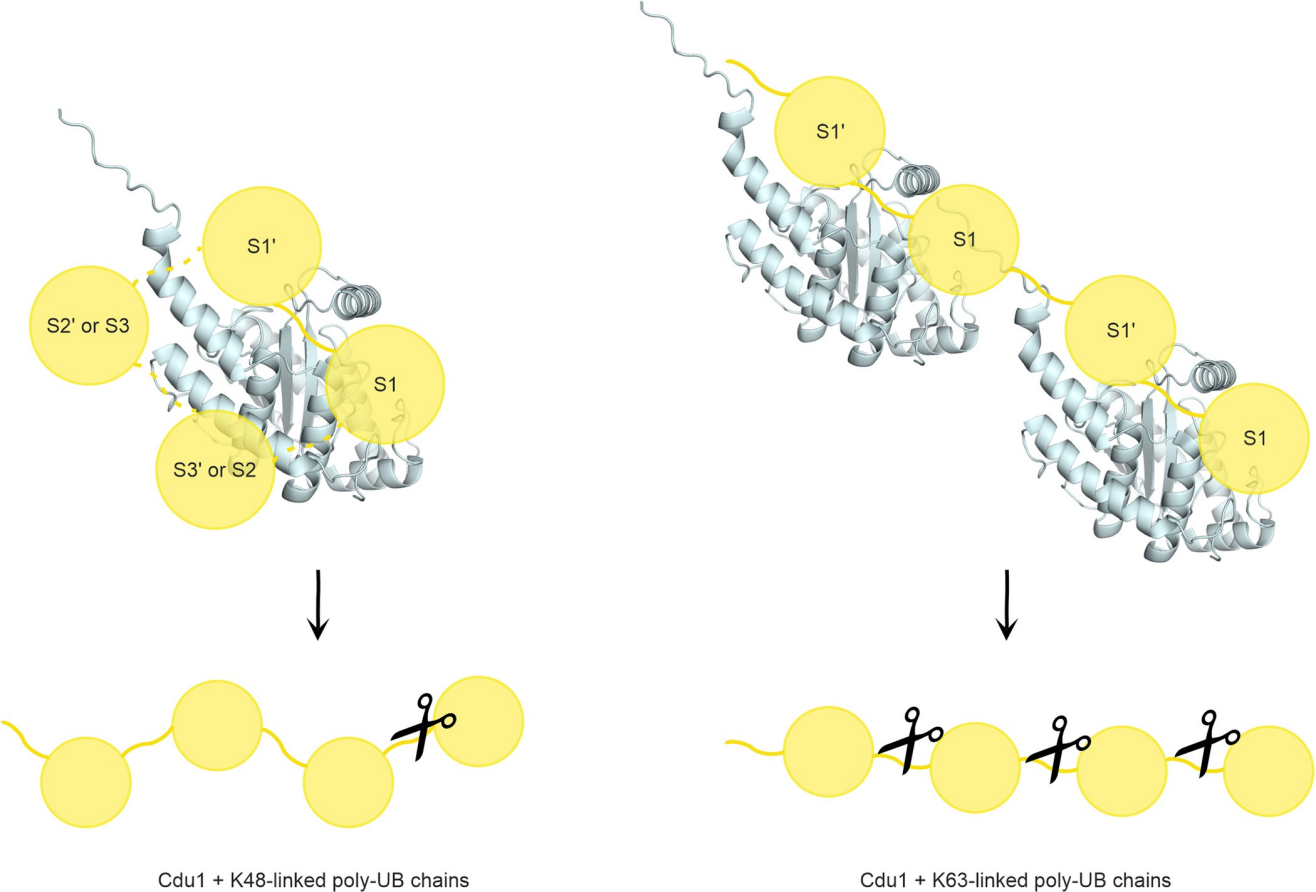
Model of the differential recognition of K48- and K63-linked poly-UB substrates by Cdu1. While K48-linked chains (left) are recognized by up to four distinct ubiquitin binding interfaces, for K63-linked chains only two sites are available.

The drastically increased activity of Cdu1 towards poly-UB substrates compared to its homologue Cdu2 prompted our analysis of the molecular basis for the substrate recognition of Cdu1. Our data indicate that the enhanced activity of Cdu1 can be attributed to an extended N-terminal α-helix, which is most likely absent or severely deformed in Cdu2. We further demonstrated that the differential recognition of K48- and K63-linked poly-UB chain substrates by Cdu1 may modulate its activity towards both chain linkages (Fig 7). Intriguingly, additional interaction interfaces for K48-linked poly-UB substrates, which were not observed for K63-linked poly-UB substrates did not result in a generally increased activity towards the former linkage. Instead, the hydrolase activity is steered towards selected isopeptide bond positions for K48-linked chains, while K63-linked poly-UB substrates are cleaved stochastically.

Based on our NMR studies and analysis of binding-deficient mutants, we propose dedicated S1- and S1’-sites on Cdu1 for K48- and K63-linked chains, and additional S- or S’-sites only for K48-linked substrates. The S1’-site is located between I337 and the catalytic C345. Distinct enthalpic and entropic contributions to the binding of K48- and K63-linked di-UB substrates indicate distinct interaction motifs for the proximal ubiquitin moiety. This likely involves M153 for K63-linked, but not K48-linked, poly-UB chains, as its mutation to alanine resulted in a clear reduction in activity for the former but not the latter substrates. CSP data for M149 and M153 with K48-linked poly-UBs, along with the adverse effects of their alanine mutations for the interaction, suggest that these residues are rather involved in the formation of a second S’-site for K48-linked chains, which only has a minor influence on substrate turnover. Lastly, the reduced affinity of the Cdu1^CA^ Q384A/A385R variant for K48-linked poly-UB substrates points to a ubiquitin interaction interface around helix αH. Cdu1’s preference for cleaving K48-linked tetra-UB chains at the distal isopeptide bond implies a third S’-site (S3’) at this position. The arrangement would be similar to that found in MINDY1/2, which has four individual S’-sites that mediate exo-cleavage of short poly-UB chains [7]. However, as helix αH is well positioned to interact with a second distal ubiquitin molecule that is conjugated via K48 to the ubiquitin bound at the S1-site, it is plausible that helix αH instead functions as a second S-site. Ultimately, the lack of available structural data unfortunately prevents the unambiguous assignment of each interaction motif.

DUB substrate selectivity remains an ongoing research topic and the search for novel DUB-ubiquitin interaction motifs is essential to understand many cellular processes. Our data provide clues towards distinct roles of Cdu1 in processes associated with *C*. *trachomatis* infection. The efficient activity against poly-UB substrates could potentially enable Cdu1 to act as a multi-purpose DUB, which would explain its multitude of known targets. The greatly reduced activity of Cdu2 towards ubiquitin chains is intriguing and rather suggests direct interactions with potential substrates of which to our knowledge none have been described so far. The differential recognition of K48- and K63-linked poly-UB substrates by Cdu1 may enable a tailored activity towards different poly-UB chains. K63-linked chains are likely more rapidly degraded but not as efficiently recognized at low abundance compared to K48-linked chains due to their decreased affinity to Cdu1. Further studies are required to fully understand the implications of the differential recognition of K48- and K63-linked poly-UB substrates during chlamydial infections.

## Materials and methods

### Cloning

For recombinant expression, Cdu1(130–401), Cdu1^Δhelix^(163–401) (Uniprot: B0B9A0) or Cdu2(69–339) (Uniprot: B0B999) were cloned into pETM-14 vectors behind the 3C cleavage site and in frame with the N-terminal 6xHis-tag using the SLIC protocol [32]. Cdu1^CH^ was generated by replacing the N-terminal helix αA (aa 130–165) in Cdu1(130–401) with the corresponding residues of Cdu2 (aa 69–91). Similarly, Cdu2^CH^ was generated by replacing the N-terminal sequence (aa 69–91) of Cdu2(69–339) with the residues of helix αA (aa 130–165) in Cdu1. Wildtype ubiquitin and variants were cloned into the pET-30a vector. Mutations in all constructs were introduced using the QuickChange site-directed mutagenesis kit according to the manufacturer’s instructions.

For mammalian cell transfection, Myc-tagged Cdu1(130–401), Cdu1^Δhelix^(163–401), Cdu1(130–401)^CA^ and Cdu1(130–401) L294R/N296G were cloned into the pcDNA3 vector. All clones were verified by DNA sequencing.

### Protein expression and purification

Rosetta 2(DE3) cells were transformed with pETM-14 plasmids bearing native or mutated 6xHis-tagged Cdu1(130–401) or Cdu2(69–339) constructs, as well as the respective inactive Cdu1 variants in which C345 was mutated to Ala (CA)) and grown at 37°C and 200 rpm in LB medium supplemented with 34 μg/mL chloramphenicol and 50 μg/mL kanamycin. At an OD_600_ of 0.8, protein expression was induced by the addition of 0.5 mM IPTG and cells were further incubated at 18°C for 20 h. Similarly, pET30a plasmids harboring tag-free mono-ubiquitin were transformed into Rosetta2(DE3) cells and expressed as described above.

For unlabeled Cdu1 variants, harvested cells were resuspended in lysis buffer (50 mM HEPES pH 8.0, 300 mM NaCl, 1 mM TCEP, supplemented with EDTA-free cOmplete protease inhibitor (Roche) and DNaseI) and lysed with a cell disruptor at 1.500 bar. The lysate was cleared by centrifugation at 54.000 x g for 1 h and subsequently applied twice to a Ni-IDA (Macherey & Nagel) gravity flow column. The beads were washed with 20 column volumes of wash buffer (50 mM HEPES pH 8.0, 1 M NaCl, 1 mM TCEP) and target proteins were subsequently eluted in elution buffer (50 mM HEPES pH 8.0, 300 mM NaCl, 1 mM TCEP, 400 mM Imidazole). HRV14 3C-protease was added to the eluate in a 1:100 ratio prior to overnight dialysis against lysis buffer. Cleaved Cdu variants were concentrated with Amicon Ultra centrifugal filters (Millipore) and applied to a Superdex 75 16/600 size exclusion chromatography (SEC) column (GE Healthcare) equilibrated with SEC buffer (20 mM HEPES pH 8.0, 150 mM NaCl, 1 mM TCEP). Fractions of sufficient purity were concentrated, aliquoted and flash-frozen in liquid nitrogen for storage at -80°C.

Cell pellets with overexpressed mono-UB variants and linear M1-linked tetra-UB were resuspended in lysis buffer and lysed by sonication. Then, perchloric acid was added to a final concentration of 0.5% (v/v) to the cleared lysate under continuous stirring and incubated for 5 min on ice until an opaque precipitate formed. After centrifugation at 4000 x g, the cleared supernatant was dialyzed overnight against 50 mM sodium acetate buffer at pH 4.5. Dialyzed samples were applied to HiTrap cation exchange columns (GE Healthcare) and ubiquitin was eluted by applying a continuous NaCl gradient with concentrations ranging from 0 to 400 mM. Pure ubiquitin fractions were pooled, buffer exchanged to SEC buffer during concentration and flash frozen in liquid nitrogen for storage at -80°C.

For NMR experiments, 6xHis-tagged wildtype or inactive (CA) Cdu1(130–401) or variants containing single amino acid mutations for peak assignment were expressed in D_2_O M9 minimal medium supplemented with 34 μg/mL chloramphenicol and 50 μg/mL kanamycin. Methyl-labeling of Ala, Ile-δ_1_, Leu, Met, Val methyl groups (AILMV-labeling) was achieved by adding 100 mg/L [^1^H, ^13^C]-ε-methionine, 60 mg/L [^1^H, ^13^C]-α-ketobutyrate and 100 mg/L [^1^H, ^13^C]-α-ketoisovalerate precursors 1 h before induction and 100 mg/L [^1^H, ^13^C]-β-alanine 20 min before induction to the culture as described previously [33]. Protein expression was induced with 1 mM IPTG and cultures were subsequently incubated for 16 h at 25°C. Harvested cells were resuspended in lysis buffer (50 mM sodium phosphate pH 7.5, 150 mM NaCl, 1 mM DTT, 15 mM imidazole supplemented with 0.1% Triton X-100). After lysis by sonication, the cleared lysate was applied to Ni-NTA beads (Machery Nagel). Beads were washed with wash buffer (50 mM sodium phosphate pH 7.5, 150 mM NaCl, 1 mM DTT, 15 mM imidazole) and eluted in elution buffer (50 mM sodium phosphate pH 7.5, 150 mM NaCl, 1 mM DTT, 300 mM imidazole). The His-tag was removed by the addition of 1:100 3C protease during dialysis against 20 mM sodium phosphate pH 7.5, 150 mM NaCl, 1 mM DTT. Next, the protein was concentrated and applied to a Superdex 75 pg SEC column (GE Healthcare) and eluted in NMR buffer (20 mM HEPES pH 7.5, 150 mM NaCl and 2mM DTT).

### Preparation of ubiquitin propargylamide (UB-PA)

A pTXB1 plasmid bearing mono-ubiquitin(1–75), C-terminally fused to intein and a chitin binding domain [34] was transformed into Rosetta 2 cells and expressed as described above for wildtype mono-UB. Harvested cells were resuspended in lysate buffer (50 mM HEPES, 100 mM sodium acetate buffer at pH 6.5) and lysed by sonication. The cleared lysate was incubated with chitin beads (New England Biolabs) for 1 h at 4°C prior to washing with lysate buffer. Ubiquitin was eluted by incubation with lysate buffer supplemented with 50 mM sodium 2-mercaptoethanesulfonate (Sigma-Aldrich) over night at room temperature. The eluate was subsequently concentrated and diluted in lysis buffer supplemented with 250 mM propargylamine (Sigma-Aldrich) and incubated for 3 h at 37°C. The formed ubiquitin propargylamide suicide probe was purified by size-exclusion chromatography using a Superdex 75 pg column (GE Healthcare) equilibrated with 20 mM HEPES pH 8.0, 150 mM NaCl, 1 mM TCEP.

### Generation of the Cdu1 ubiquitin-propargylamide adduct

Native or ^1^H, ^13^C-AILMV-methyl labeled Cdu1 was incubated with either a 1.5- or 4-fold molar excess of UB-PA, respectively, for 24 h at 4°C in the presence of 20 mM HEPES pH 8.0, 150 mM NaCl, 1 mM TCEP. The formed adducts were purified by size-exclusion chromatography using a Superdex 75 pg column (GE Healthcare) pre-equilibrated with 20 mM HEPES pH 8.0, 150 mM NaCl, 1 mM TCEP.

### In vitro ubiquitin chain formation

In vitro ubiquitin chain formation reactions were performed as previously described [35,36]. For K48-linked chains, 2.8 mM mono-ubiquitin were supplemented with 1 μM Uba1 and 25 μM Cdc34 in ddH_2_O, containing 10 mM MgCl_2_. Similarly, K63-linked chains were prepared by supplementing 1.4 mM mono-ubiquitin with 1 μM Uba1, 8 μM Ubc13 and 8 μM Mms2 in the presence of 10 mM MgCl_2_. K11-linked chains were generated by incubating 2.94 mM mono-UB^K63R^ with 1 μM Uba1, 10 μM Ube2S-UBD in 40 mM Tris-HCl pH 7.5, 10 mM MgCl_2_. Chain formation reactions were started by the addition of 10 mM ATP and incubated for 3–4 h at 37°C and subsequently stopped by a 25-fold dilution into 50 mM sodium acetate pH 4.5. Ubiquitin chains of various lengths were separated by ion exchange chromatography using a 1 mL Resource S column (GE Healthcare) with an elution gradient ranging from 0 mM to 400 mM NaCl.

### Preparation of poly-ubiquitin substrates fluorescein-labeled at the distal ubiquitin

200 μM fluorescein-maleimide (FAM) (Lumiprobe) were added to 100 μM mono-UB containing either S20C/K48R or S20C/K63R mutations in 20 mM HEPES pH 7.0, 150 mM NaCl, 2 mM TCEP. The labeling reaction was incubated for 5 min at 4°C and subsequently quenched by the addition of 50 mM DTT, resulting in approximately 90% labeled mono-UB. Unreacted FAM-maleimide was removed by size-exclusion chromatography using a Superdex 75 10/300 increase column (GE Healthcare) equilibrated in SEC buffer (20 mM HEPES pH 8.0, 150 mM NaCl, 1 mM TCEP).

Chain formation reactions were performed similarly as described above. For K48-linked chains, 400 μM wildtype K48-linked tri- or tetra-UBs, respectively, were incubated with 800 μM FAM-labeled S20C/K48R mono-UB, 1 μM Uba1 and 15 μM Cdc34 in the presence of 10 mM MgCl_2_ and 10 mM ATP. Labeled K63-linked chains were assembled by incubation of 300 μM native K63-linked tri- or tetra-UBs, respectively, with 600 μM FAM-labeled S20C/K63R mono-UB, 0.5 μM Uba1, 4 μM Ubc13 and 4 μM Mms2 in the presence of 10 mM MgCl_2_ and 10 mM ATP. All reactions were incubated at 37°C for 4 h prior to a 25-fold dilution into 50 mM sodium acetate buffer pH 4.5. FAM-labeled chains were separated by ion exchange chromatography as described above and buffer exchanged to 20 mM HEPES pH 8.0, 150 mM NaCl, 1 mM TCEP.

### UB-Rho110Gly DUB activity assay

In the presence of 20 mM HEPES pH 7.5, 50 mM NaCl, 1 mM TCEP, and 50 μg/mL BSA, 20 μL Cdu variants at concentrations of 0.6 nM were added to 20 μL of a 2-fold titration series of UB-Rho110Gly (UbiQ) with final concentrations ranging from 0.03 μM to 4 μM. The time dependent increase in fluorescence (Ex: 487 nm; Em: 535 nm) upon UB-Rho110Gly cleavage was measured in a 384 well plate at 28°C using a ClarioStar plate reader (BMG Labtech). The initial constant phase of the reaction was fitted to a linear equation using the MARS analysis software (BMG Labtech) The enzymatic activity was plotted against the corresponding substrate concentrations and the Michaelis-Menten equation was subsequently fitted to the obtained data in GraphPad Prism. Uncertainties in the fit parameters were estimated by Monte-Carlo simulation using MATLAB (The MathWorks Inc).

### Ubiquitin chain cleavage assay

Native or FAM-labeled, homotypic M1-, K11-, K48- or K63-linked ubiquitin substrates at concentrations of 2.5 μM or 5 μM, respectively, were incubated with 25 nM Cdu variants. For FAM-labeled K63-linked substrates only 12.5 nM enzyme was used. All cleavage reactions were performed at 28°C in 20 mM HEPES pH 8.0, 150 mM NaCl and 1 mM TCEP and were subsequently stopped at the indicated time points by the addition of SDS loading buffer to the reaction samples. For control samples, the respective ubiquitin chains and enzymes were directly mixed in SDS loading buffer. All samples were applied to 4–20% SDS gradient gels and subsequently silver stained using the Pierce silver stain kit (Thermo Scientific) according to the manual provided. The fluorescence signals of the FAM-labeled bands were visualized on a Typhoon FLA 7000 scanner (GE Healthcare) prior to silver staining. The scanned gels were quantified with ImageJ [37] by determining the background subtracted fluorescence signals and gray scale values. For each reaction time point, the proportion of fluorescent to total amount of ubiquitin in each product band was calculated and compared.

### Native polyacrylamide gel electrophoresis (native PAGE)

5% native PAGE gels were prepared by mixing 4.2 mL of 30% polyacrylamide mix (Roth) with 1.25 mL of 10 X running buffer (250 mM Tris, 1.92 M glycine), 19.5 mL ddH_2_O, 175 μL APS and 25 μL TEMED. Cdu complexes with poly-UB were pre-formed at 28°C by the addition of 250 μM of the respective Cdu1^CA^ variants to 50 μM of either K48- or K63-linked poly-UB chains to a final 2:1 stoichiometry and separated at room temperature applying a constant voltage of 90 V in 1 X running buffer and subsequently stained with Coomassie G250.

### Differential scanning fluorimetry

In 20 mM HEPES pH 8.0, 150 mM NaCl and 1 mM TCEP, Cdu variants at concentrations of 4 μM in the presence of 0.1% SYPRO orange (Invitrogen) were incubated at increasing temperatures ranging from 25°C to 90°C with a 1°C/min gradient using a Stratagene Mx3005P (Agilent Technologies) thermocycler. The SYPRO orange fluorescence was normalized and plotted as a function of temperature and the T_M_ value was determined at the minimum of the first derivative of the measured fluorescence.

### Circular dichroism spectroscopy

The circular dichroism of Cdu1 variants in PBS at concentrations of 2.5 μM was measured using a J-810 spectropolarimeter (Jasco) at 25°C. For each variant five consecutive scans from 195 nm to 300 nm were averaged and buffer subtracted.

### Isothermal titration calorimetry

A MicroCal ITC200 microcalorimeter (Malvern Panalytical) was used to analyze the interaction of M1-, K11-, K48- and K63-chains with Cdu1^CA^ variants. In 20 mM HEPES pH 8.0, 150 mM NaCl, 1 mM TCEP, 20 μM of the respective poly-UBs were titrated with 200 μM Cdu1^CA^ variants at 28°C with a single 1.2 μL and 15 consecutive injections of 2.4 μL under continuous stirring at 600 rpm. Due to the elevated binding stoichiometries, 400 μM of the respective Cdu1^CA^ variants were used with K63-linked poly-UB chains exceeding tri-UB. For the Cdu1^CA^ double mutants, 30 μM K48-linked poly-UBs and 300 μM Cdu1^CA^ variants were used instead. To measure the interaction of Cdu1^CA^ with mono-UB, 200 μM DUB were titrated with 2 mM mono-UB. The measured thermograms were integrated using the Microcal-ITC-ORIGIN analysis software (Malvern Panalytical) and obtained isotherms were fitted to a one set of sites binding model.

### Nuclear magnetic resonance (NMR) spectroscopy

Chemical shift perturbation (CSP) experiments were performed by recording 2D ^1^H,^13^C-methyl-SOFAST-HMQC spectra of 45 μM AILMV-labeled inactive (CA) Cdu1 or wildtype Cdu1 covalently modified with UB-PA in the absence and presence of increasing amounts of unlabeled monomeric ubiquitin or K48- or K63-linked tetra-UB chains. For resonance assignment of the Cdu1 methyl groups 14 individual point mutants (M149V, I159V, A165G, I170V, I186V, I196V, I204V, I219V, I225V, M262V, I280V, M299V, I331V and I337V) were generated and their 2D ^1^H,^13^C-methyl-HMQC spectra compared to the Cdu1^CA^ spectrum. Additionally, SOFAST-HMQC-based 3D CCH NOESY spectra were recorded on AILMV-labeled Cdu1^CA^ with mixing times of 300 ms and 50 ms, respectively [38]. CSP analysis was performed by quantifying the signal intensities for the peak positions of the apo protein in the reference (apo) spectra (I_ref_) and the titration spectra (I). To map the CSPs on the Cdu1~UB-PA structure (PDB-ID: 2GZS) the relative decrease in signal intensity (I / I_ref_) at the respective peak position of the apo protein was calculated and residues with a loss of signal intensity of more than 85% color coded with a color gradient ranging from 85–100%.

All NMR measurements were conducted in NMR buffer with 10% (v/v) D_2_O at 25°C, on Bruker 500 MHz, 600 MHz and 800 MHz Avance Neo spectrometers. All NMR data were acquired using TOPSPIN 4.0.2 (Bruker Biospin GmbH), processed with the NMRPipe/NMRDraw program suite [39], analyzed and visualized with XEASY [40] or NMRFAM-Sparky [41].

### Size exclusion chromatography coupled small angle X-ray scattering (SEC-SAXS)

Cdu1^CA^ complexes with K48- and K63-linked poly-UB chains were assembled at a concentration of 170 μM in 20 mM HEPES pH 8.0, 150 mM NaCl, 1 mM TCEP at a 1:1 stoichiometry. SAXS data were measured at the BM29 bioSAXS beamline at the ESRF [42]. Apo Cdu1, Cdu1~UB-PA and the pre-assembled complexes were applied to a Superdex 75 10/300 increase (GE healthcare) column and in each case 1500 two second frames were taken on a Pilatus 2M detector. The data were reduced with the automated pipeline, FreeSAS [43]. The software Scatter [30] was then used for peak selection and background subtraction and analysis. 3D bead models were formed using the program DAMMIF [44]. Data fitting of the crystal structure with the PDB entry 5HAG to the scattering data for apo Cdu1 was calculated with the program FoXS [45].

### In vivo IκBα stability assay

2 x 10^5^ HEK293T cells (ATCC CRL-3216, RRID:CVCL_0063) incubated in high glucose DMEM (Sigma-Aldrich) supplemented with 10% (v/v) heat-inactivated FBS (Gibco) at 37°C and 5% (v/v) CO_2_ were seeded per well of 12 well plates. For the transfection, cells were treated with 25 μM chloroquine prior to the addition of the transfection reagent consisting of a 1:1 mixture of 2 μg plasmid DNA in 250 mM CaCl_2_ and 2×HBS (50 mM HEPES, 140 mM NaCl, 1.5 mM Na_2_HPO_4_, pH 7.05).

One day post transfection, cells were treated with 30 ng/ml recombinant human TNF-α (Peprotech) for the indicated times. TNFα treated cells were lysed in SDS sample buffer and separated by SDS-PAGE. Transfer to PVDF membranes was performed with the Trans-Blot Turbo system (BioRad) at 1.3 A per mini gel for 7 min. Membranes were blocked in 5% (w/v) bovine serum albumin (BSA) in Tris-buffered saline supplemented with 0.05% Tween-20 (TBS-T) and incubated with IκBα (Cell Signaling Technology), Myc-tag (Cell Signaling Technology) and β-Actin (Sigma) primary antibodies. Proteins were detected with HRP-coupled goat anti-rabbit IgG (Jackson ImmunoResearch) or mouse-IgGκ binding protein (Santa Cruz Biotechnology) secondary antibodies using self-made enhanced chemiluminescence (ECL) solutions (ECL solution 1: 100 mM Tris-HCl pH 8.6, 2.5 mM luminol, 0.4 mM p-coumaric acid; ECL solution 2: 100 mM Tris-HCl pH 8.6, 0.02% (v/v) H_2_O_2_) and an Intas Chemiluminescence Imager. Quantification was performed using the ImageJ software and statistical significance was calculated using a one-tailed t-test.

## Supporting information

S1 Table Binding parameters for the interactions of Cdu1^CA^ with poly-UB substrates. (DOCX)

S2 Table Parameters of the SAXS beamline and data processing. (DOCX)

S1 Fig **A:** Secondary structure prediction of Cdu2 (Uniprot: B0B999) from *C*. *trachomatis* by JPred [27]. The existence of helix αA of Cdu1 is not predicted for Cdu2. **B:** Structure prediction by Alphafold [28] of full length Cdu2. The confidence of the predicted model is color-coded. An N-terminal alpha helix in Cdu2 is only predicted with low confidence and with a conformation that is distinct from the one observed in Cdu1.
(TIF)

S2 Fig Protein integrity of selected DUB variants. **A:** Differential scanning fluorimetry with selected Cdu variants (n = 3). The given T_M_ values are defined as the temperature values at the inflection point of the unfolding curve. **B:** Circular dichroism spectroscopy of selected Cdu1 variants (n = 1).
(TIF)

S3 Fig **A:** ITC experiments in which 20 μM of the indicated poly-UB chains were titrated with 200 μM Cdu1^CA Δhelix^ (n = 2). **B:** ITC experiments in which 20 μM K48- and K63-linked tetra-UB was titrated to 200 μM of Cdu1^CA^ I225A/I267R (n = 1). **C:** ITC experiments in which 2 mM mono-UB was titrated to 200 μM of Cdu1^CA^ (n = 3).
(TIF)

S4 Fig **A:** ITC experiments in which 20 μM of the indicated poly-UB chains were titrated with 200 μM Cdu1^CA^ (n = 2). **B:** Cdu1 cleavage assay of Cdu1 with M1- and K11-linked tetra-UB substrates (n = 2). **C:** Native PAGE of 100 μM Cdu1 and Cdu2 complexes with K48-linked and K63-linked poly-UB chains at constant amounts of mono-UB within each sample (n = 2). Final poly-UB concentrations amount to 200 μM mono-UB, 100 μM di-UB, 66.7 μM tri-UB and 50 μM tetra-UB.
(TIF)

S5 Fig **A & B:** FAM-labeled poly-UB cleavage assay of Cdu1 with K48-linked (**A**) and K63-linked (**B**) penta-UB. *Upper panel*: Silver staining. *Lower panel*: Fluorescence signal. For the quantification, colored bars indicate the fraction of fluorescence within each reaction product species at the specified time points. Black bordered bars indicate the fraction of total ubiquitin measured after silver staining (n = 3). **C:** ITC experiments in which 20 μM of the FAM-labeled K48- or K63-linked poly-UB substrates were titrated with 200 μM Cdu1^CA^ (n = 2).
(TIF)

S6 Fig **A:** Calculated P(r) distributions of SEC-SAXS data generated with isolated poly-UB chains (left) **B:** Kratky-Plot for the SEC-SAXS samples. **C:** DAMMIF *ab initio* modeling with data generated from SEC-SAXS experiments with apo Cdu1, Cdu1~UB-PA and Cdu1^CA^ complexes formed with either K48-linked di-, tri- and tetra-UB or K63-linked tetra-UB respectively. The crystal structure of Cdu1 (PDB: 5HAG) was placed into the apo model.
(TIF)

S7 Fig Overlay of selected regions of the 2D ^1^H,^13^C-correlation spectra of AILMV-labeled Cdu1^WT^ conjugated to UB-PA in the absence (black) and in the presence of a 12-fold excess of monomeric ubiquitin (red). (TIF)

S8 Fig FAM-labeled poly-UB chain cleavage assay for Cdu1 M149A/M153A and Cdu1 Q384A/A385R with K48-linked and K63-linked tetra-UB distally labeled with fluorescein. *Top*: Silver staining. *Bottom*: Fluorescence signal. For the quantification, colored bars indicate the fraction of fluorescence within each reaction product species at the specified time points. Black bordered bars indicate the fraction of total ubiquitin measured after silver staining (n = 3).
(TIF)
