# Supplementary material for: Linkage-specific ubiquitin binding interfaces modulate the activity of the chlamydial deubiquitinase Cdu1 towards poly-ubiquitin substrates
Source: PLoS Pathog. 2024 Oct 21;20(10):e1012630. doi: 10.1371/journal.ppat.1012630 (PMC11527256; doi:10.1371/journal.ppat.1012630)
Supplement: S1 Table — (DOCX) [file ppat.1012630.s001.docx]

**Table S1: Binding parameters for the interactions of Cdu1^CA^ with poly-UB substrates.**

|  |  |  | K_D_ [µmol*L^-1^] | N | ∆H [cal*mol^-1^] | ∆S [cal*mol^-1^*deg^-1^] |
| --- | --- | --- | --- | --- | --- | --- |
| Native | M1 | tetra-UB | n.d. | n.d. | n.d. | n.d. |
|  | K11 | di-UB | 4.63 (± 1.44) | 0.71 (± 0.1) | 2447 (± 251) | 32.6 (± 0.2) |
|  |  | tri-UB | 1.41 (± 0.34) | 1.01 (± 0.14) | 1516 (± 301) | 31.9 (± 0.5) |
|  |  | tetra-UB | 3.30 (± 2.06) | 0.88 (± 0.06) | 2203 (± 64) | 32.6 (± 1.1) |
| FAM | K48 | tetra-UB | 0.38 (± 0.05) | 1.0 (± 0.02) | 10820 (± 42) | 65.3 (± 0.4) |
|  | K63 | tetra-UB | 5.95 (± 0.54) | 1.67 (± 0.05) | 11075 (± 176) | 60.7 (± 0.4) |
