# Supplementary material for: Linkage-specific ubiquitin binding interfaces modulate the activity of the chlamydial deubiquitinase Cdu1 towards poly-ubiquitin substrates
Source: PLoS Pathog. 2024 Oct 21;20(10):e1012630. doi: 10.1371/journal.ppat.1012630 (PMC11527256; doi:10.1371/journal.ppat.1012630)
Supplement: S2 Table — (DOCX) [file ppat.1012630.s002.docx]

**Table S2: Parameters of the SAXS beamline and data processing.**

| Data-collection parameters |  |
| --- | --- |
| Instrument: | ESRF BM29 |
| Wavelength [Å] | 0.99 |
| q-range [Å^-1^] | 0.007-0.5 |
| Sample-to-detector distance [m] | 2.81 |
| Concentration range [mg/mL] | 1-6 |
| Temperature [K] | 293 |
| Detector | Pilatus P3-2M |
| Flux [photons/s] | 1.4*10^12^/1*10^13^ |
| Beam size at sample [µm] | 500*200 |
| software employed |  |
| Primary data reduction: | FreeSAXS, BM29 autoprocessing pipeline |
| Data processing | ScatterIV, FoXS, DAMMIF |
