# Supplementary figures and images for: Linkage-specific ubiquitin binding interfaces modulate the activity of the chlamydial deubiquitinase Cdu1 towards poly-ubiquitin substrates
Source: PLoS Pathog. 2024 Oct 21;20(10):e1012630. doi: 10.1371/journal.ppat.1012630 (PMC11527256; doi:10.1371/journal.ppat.1012630)

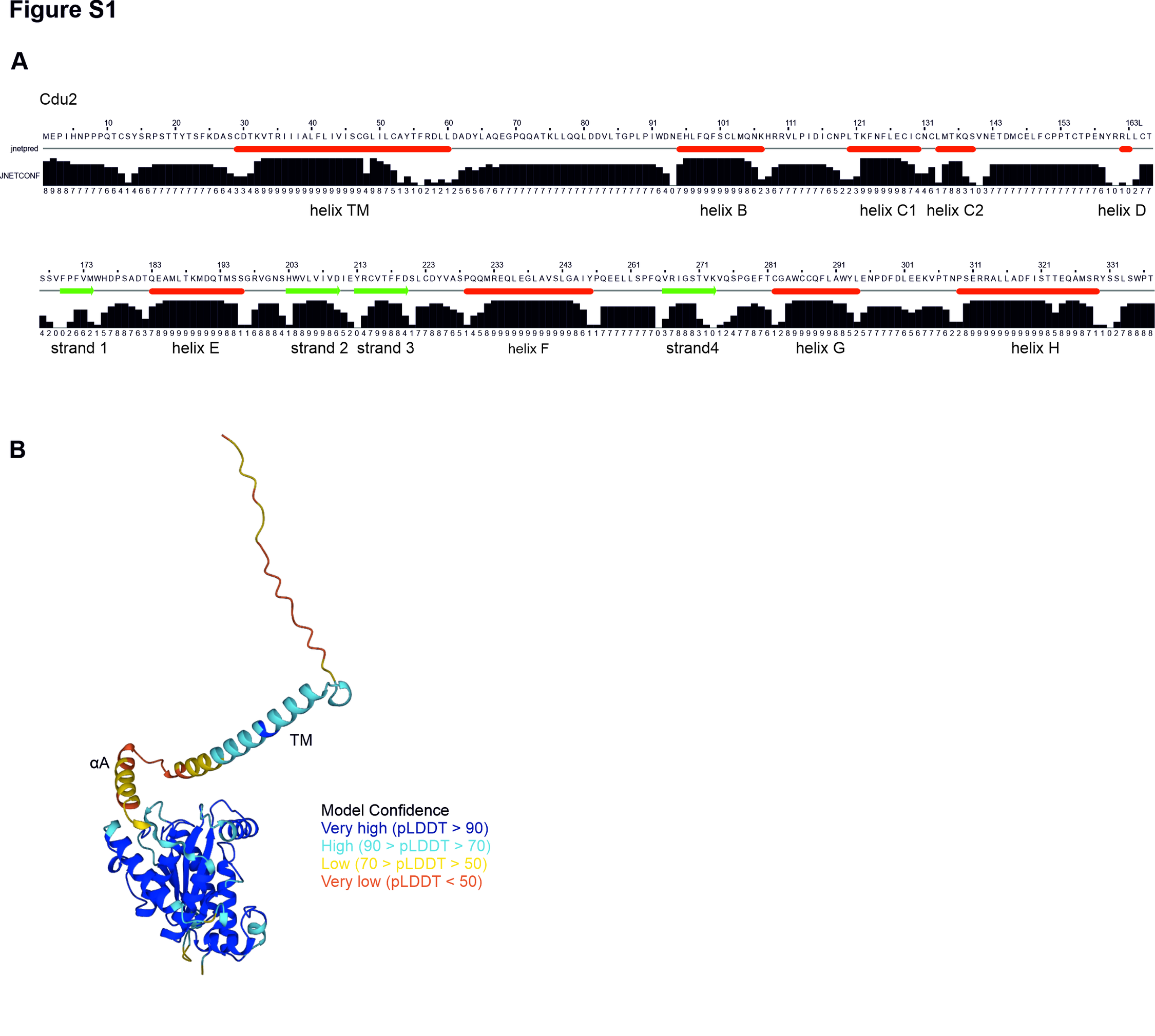

Supplement: S1 Fig — A: Secondary structure prediction of Cdu2 (Uniprot: B0B999) from C. trachomatis by JPred [27]. The existence of helix αA of Cdu1 is not predicted for Cdu2. B: Structure prediction by Alphafold [28] of full length Cdu2. The confidence of the predicted model is color-coded. An N-terminal alpha helix in Cdu2 is only predicted with low confidence and with a conformation that is distinct from the one observed in Cdu1. (TIF) [file ppat.1012630.s003.tif]

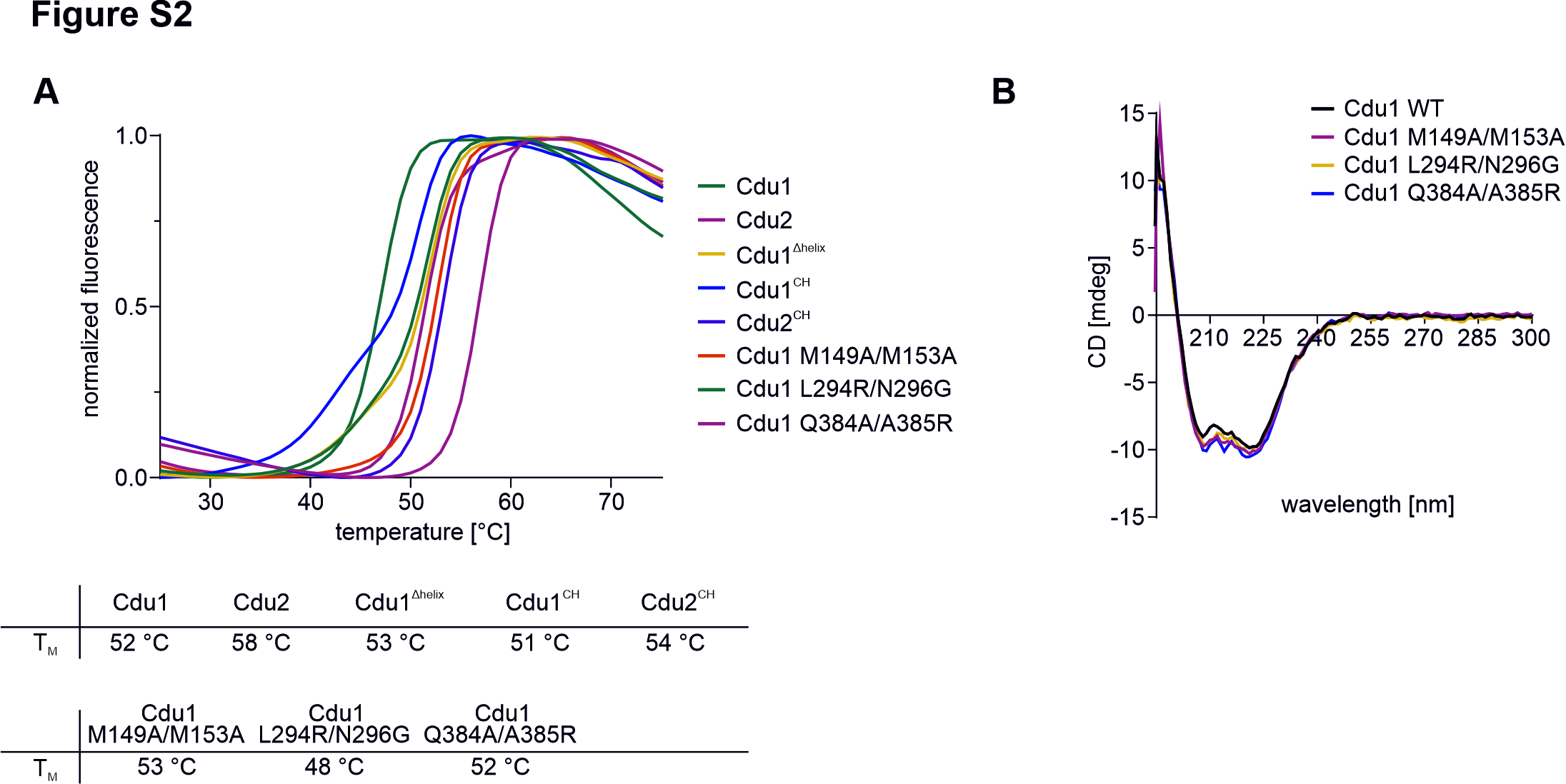

Supplement: S2 Fig — A: Differential scanning fluorimetry with selected Cdu variants (n = 3). The given TM values are defined as the temperature values at the inflection point of the unfolding curve. B: Circular dichroism spectroscopy of selected Cdu1 variants (n = 1). (TIF) [file ppat.1012630.s004.tif]

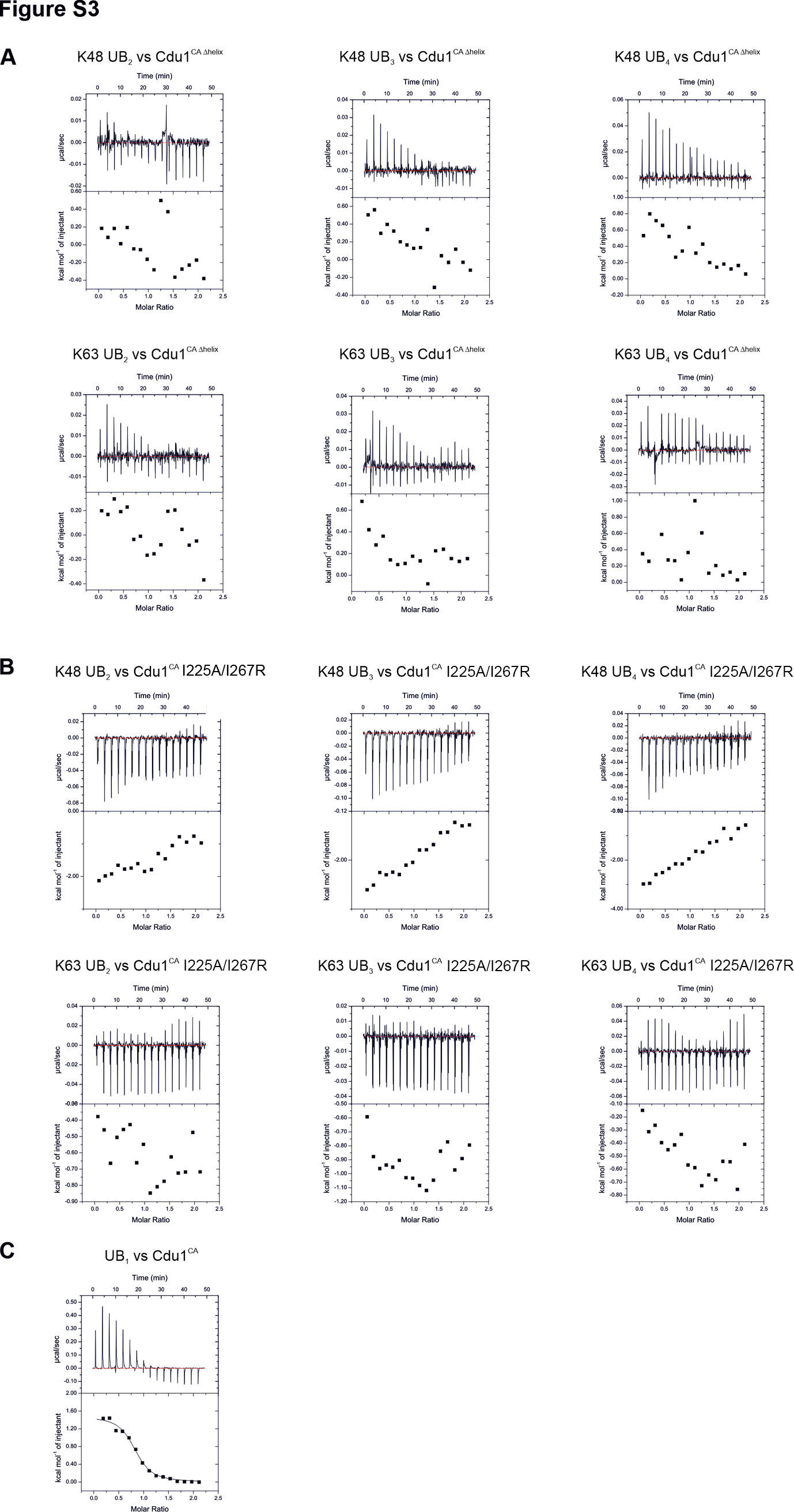

Supplement: S3 Fig — A: ITC experiments in which 20 μM of the indicated poly-UB chains were titrated with 200 μM Cdu1CA Δhelix (n = 2). B: ITC experiments in which 20 μM K48- and K63-linked tetra-UB was titrated to 200 μM of Cdu1CA I225A/I267R (n = 1). C: ITC experiments in which 2 mM mono-UB was titrated to 200 μM of Cdu1CA (n = 3). (TIF) [file ppat.1012630.s005.tif]

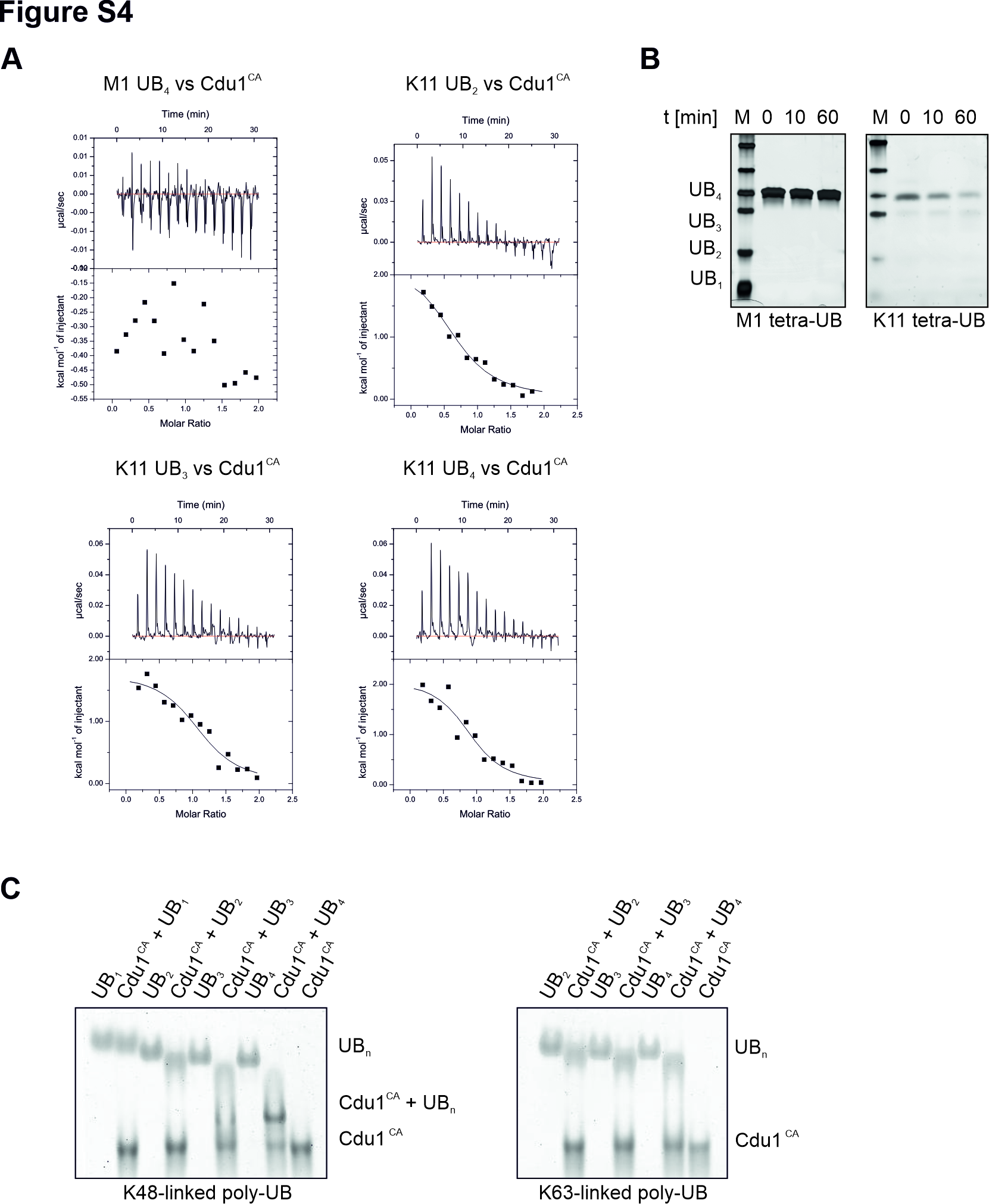

Supplement: S4 Fig — A: ITC experiments in which 20 μM of the indicated poly-UB chains were titrated with 200 μM Cdu1CA (n = 2). B: Cdu1 cleavage assay of Cdu1 with M1- and K11-linked tetra-UB substrates (n = 2). C: Native PAGE of 100 μM Cdu1 and Cdu2 complexes with K48-linked and K63-linked poly-UB chains at constant amounts of mono-UB within each sample (n = 2). Final poly-UB concentrations amount to 200 μM mono-UB, 100 μM di-UB, 66.7 μM tri-UB and 50 μM tetra-UB. (TIF) [file ppat.1012630.s006.tif]

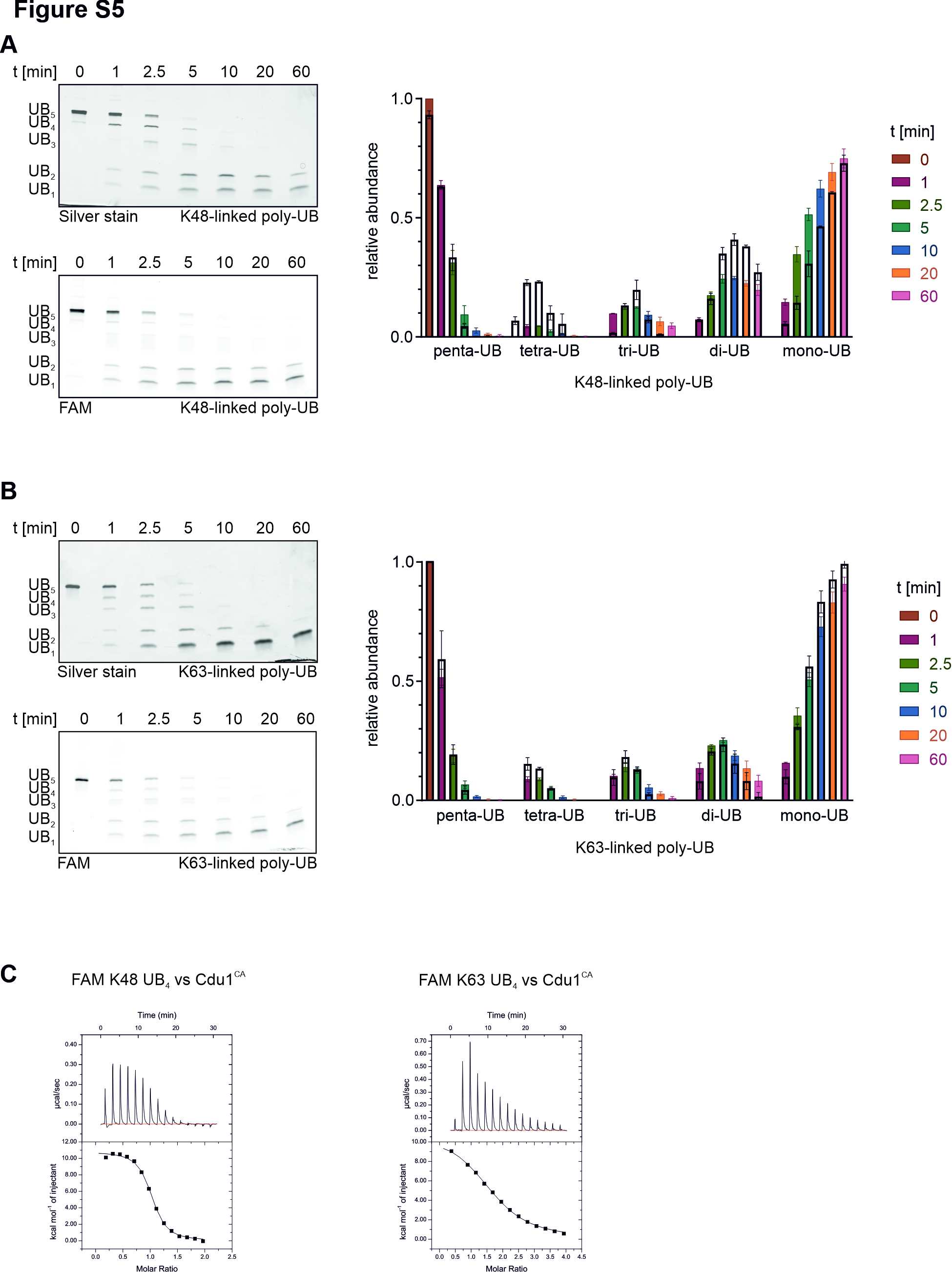

Supplement: S5 Fig — A & B: FAM-labeled poly-UB cleavage assay of Cdu1 with K48-linked (A) and K63-linked (B) penta-UB. Upper panel: Silver staining. Lower panel: Fluorescence signal. For the quantification, colored bars indicate the fraction of fluorescence within each reaction product species at the specified time points. Black bordered bars indicate the fraction of total ubiquitin measured after silver staining (n = 3). C: ITC experiments in which 20 μM of the FAM-labeled K48- or K63-linked poly-UB substrates were titrated with 200 μM Cdu1CA (n = 2). (TIF) [file ppat.1012630.s007.tif]

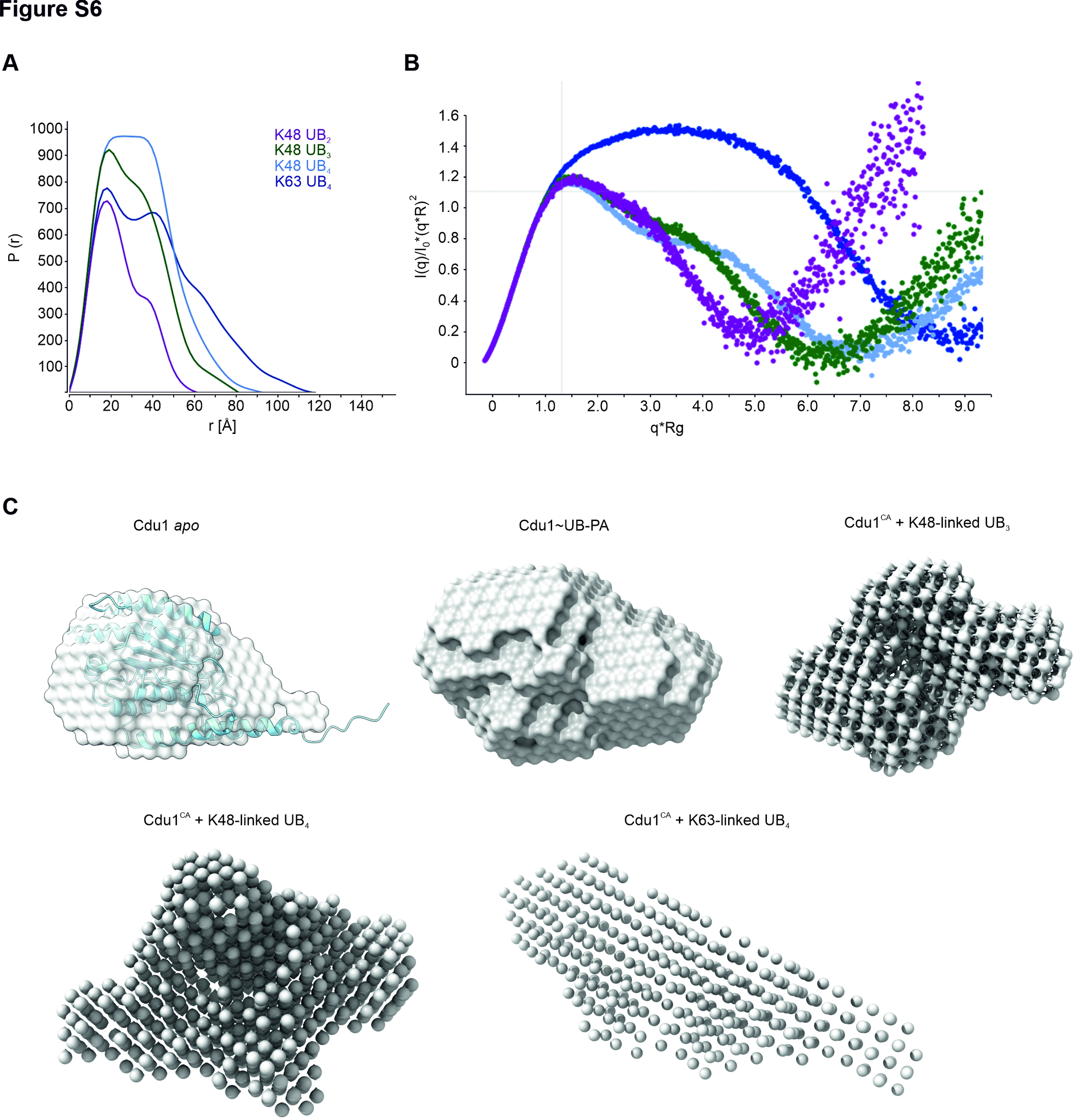

Supplement: S6 Fig — A: Calculated P(r) distributions of SEC-SAXS data generated with isolated poly-UB chains (left) B: Kratky-Plot for the SEC-SAXS samples. C: DAMMIF ab initio modeling with data generated from SEC-SAXS experiments with apo Cdu1, Cdu1~UB-PA and Cdu1CA complexes formed with either K48-linked di-, tri- and tetra-UB or K63-linked tetra-UB respectively. The crystal structure of Cdu1 (PDB: 5HAG) was placed into the apo model. (TIF) [file ppat.1012630.s008.tif]

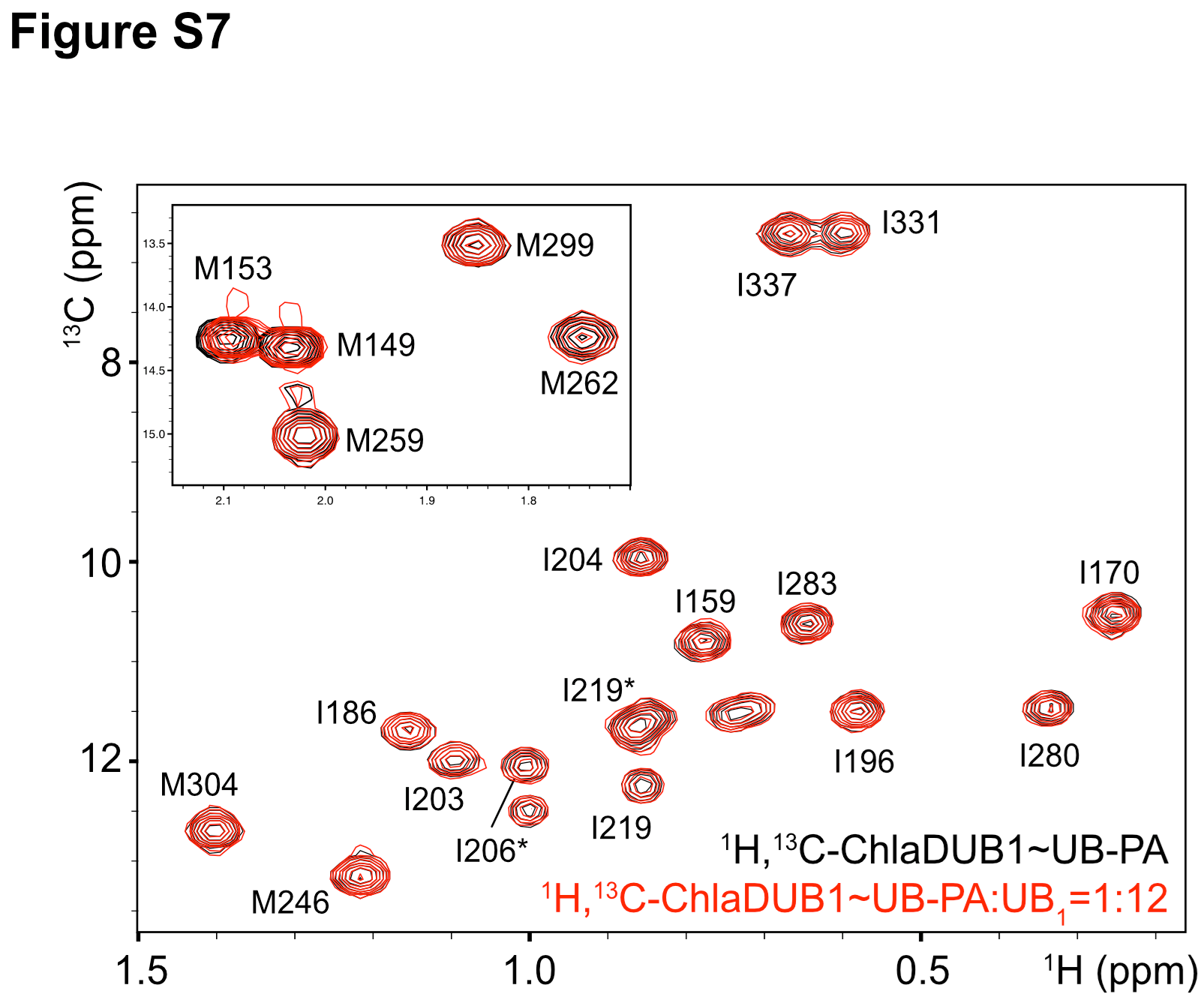

Supplement: S7 Fig — (TIF) [file ppat.1012630.s009.tif]

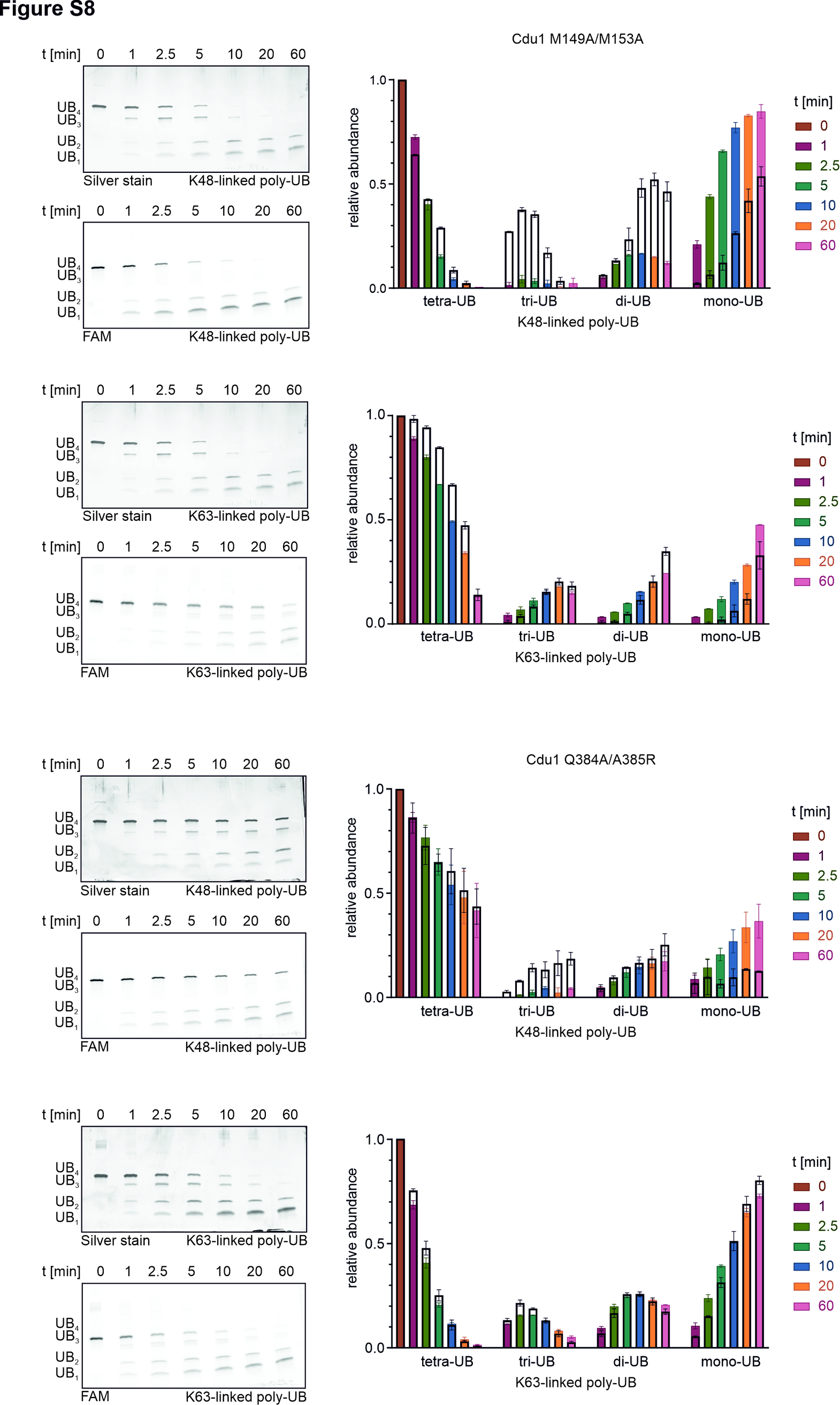

Supplement: S8 Fig — Top: Silver staining. Bottom: Fluorescence signal. For the quantification, colored bars indicate the fraction of fluorescence within each reaction product species at the specified time points. Black bordered bars indicate the fraction of total ubiquitin measured after silver staining (n = 3). (TIF) [file ppat.1012630.s010.tif]
